# Embryo-derived extracellular vesicles and their potential as biomarkers for embryo quality during IVF: a systematic review

**DOI:** 10.1093/humupd/dmag014

**Published:** 2026-05-21

**Authors:** Genevieve Boom, Vanessa Jordan, Lawrence W Chamley, Lynsey Cree

**Affiliations:** Department of Obstetrics, Gynaecology, and Reproductive Sciences, The University of Auckland, Auckland, New Zealand; Department of Obstetrics, Gynaecology, and Reproductive Sciences, The University of Auckland, Auckland, New Zealand; Department of Obstetrics, Gynaecology, and Reproductive Sciences, The University of Auckland, Auckland, New Zealand; Department of Obstetrics, Gynaecology, and Reproductive Sciences, The University of Auckland, Auckland, New Zealand; Fertility Associates, Auckland, New Zealand

**Keywords:** embryo, extracellular vesicle, IVF, IVP, embryo quality, biomarker

## Abstract

**BACKGROUND:**

Over the past decade, extracellular vesicles (EVs) released from embryos have received increasing attention for their potential role in influencing embryo–maternal communication events during implantation. Several studies have explored the relationship between embryo-derived EVs and embryo developmental competence. To synthesize current insights, we conducted a systematic review of the literature, highlighting key findings and evaluating how embryo EV profiles relate to embryo developmental competence.

**OBJECTIVE AND RATIONALE:**

A systematic review was conducted in accordance with the PRISMA guidelines. The review intended to (i) synthesize the existing knowledge on embryo-derived EV concentration, size, and cargo and their association with embryo developmental competence in mammalian systems, (ii) propose recommendations for future research, and (iii) assess the potential impact of the current literature on embryo selection during IVF.

**SEARCH METHODS:**

A literature search was performed across five databases (MEDLINE, EMBASE, BIOSIS Previews, Web of Science, and Scopus), involving keywords ‘Embryo’ AND ‘Extracellular vesicle’; many synonyms were included for each keyword along with map terms when available. The search was conducted on 14 January 2026. References were screened for additional relevant records. Abstract-only authors were contacted to determine whether full-text was available. The inclusion criteria were ‘collection of culture media from embryos of any mammalian species and isolation and assessment of EVs from it’. Reasons for exclusion included: ‘No collection of embryo culture media’, ‘No characterisation or assessment of EVs’, ‘Not embryo-derived EVs’, and ‘Review/Theory/Lacking primary data’.

**OUTCOMES:**

Our searches collected a total of 15 620 records; of these, 32 studies from 34 records were included in the systematic review. The included studies were divided into two groups: (i) studies of embryonic EV characteristics and (ii) studies that investigated the association of embryonic EV characteristics and embryo developmental competence, including embryo quality assessments based on morphology, ploidy, and pregnancy. Wide variations in embryo EV concentration and size were reported, largely due to differences in study designs. Overall, the quality of the existing literature ranged from low to moderate quality. Among the three human and eight bovine studies that examined the association of embryo EV concentration, size, and/or cargo with developmental competence, the certainty of the evidence for all outcomes was consistently very low. Studies often reported opposing directions of effects, whilst others showed no clear association. Inconsistencies in EV isolation techniques, timing of EV sampling, and methods for embryo quality assessments complicate efforts to assess whether embryonic EVs can be used as a reliable biomarker.

**WIDER IMPLICATIONS:**

Current evidence does not support the use of embryo EVs as biomarkers during IVF procedures. To enhance reproducibility and the production of high-quality studies, it is recommended that studies incorporate standardized approaches to EV isolation and characterization to align with best practices. In addition, the timing of EV sampling should be standardized, appropriate media controls used, and optimal measures of developmental competence, such as live birth, reported. Adherence to these recommendations will enable future studies to build constructively on the existing literature, with the possibility of informing clinical applications that improve patient outcomes.

**REGISTRATION NUMBER:**

https://doi.org/10.17605/OSF.IO/TPXVU

## Graphical Abstract

**Graphical Abstract:**
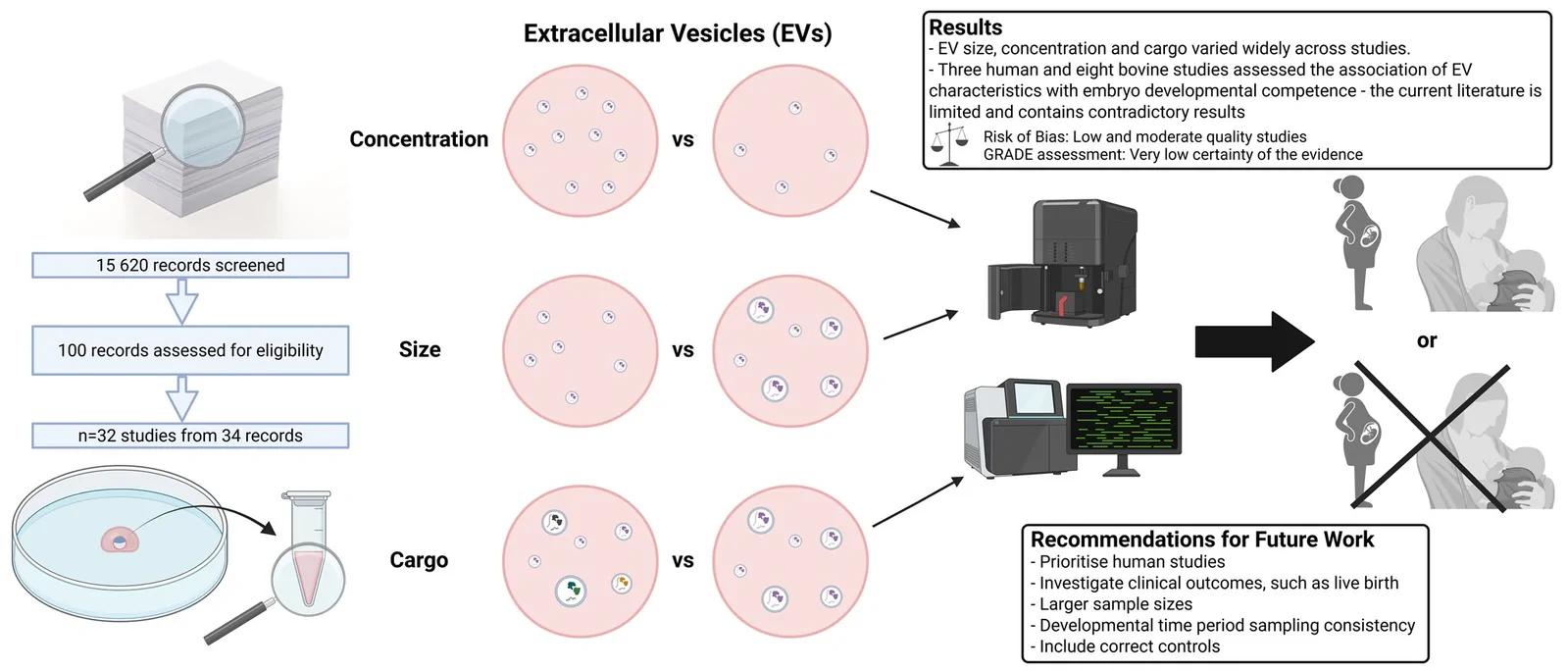
**The potential use of embryo-derived extracellular vesicles as a biomarker for embryo developmental competence remains unclear; future high-quality work focused on clinical outcomes is required.** Created in BioRender. Boom (2026) https://BioRender.com/m86o277

## Introduction

Extracellular vesicles (EVs) are lipid-bound nanoparticles containing cellular contents that are released by all cells studied to date (Yáñez-Mó *et al.*, 2015; Kalra *et al.*, 2016; Kumar *et al.*, 2024). EVs have been implicated in both physiological and pathological processes through their role in cell-to-cell communication by delivering cargos to recipient cells. The content of EVs may reflect their cell of origin and can be found in biological fluids such as blood, urine, and in embryo/cell culture medium (Pisitkun *et al*., 2004; Caby *et al.*, 2005; Giacomini *et al.*, 2017). As a result, over the past two decades, research on EVs has increased dramatically, as evidence for their role in both health and disease, as well as their potential use as biomarkers, continues to expand (Reviewed in Yates *et al.*, 2022; Kumar *et al.*, 2024).

As the field of EVs is rapidly evolving, nomenclature for EVs has varied, but the Minimal Information for Studies of Extracellular Vesicles (MISEV) guideline and periodic updates issued by the International Society for Extracellular Vesicles (Lötvall *et al.*, 2014; Théry *et al.*, 2018; Welsh *et al.*, 2024) recommend that EVs are categorized based on their size as either large EVs (>200 nm, previously referred to as micro EVs or micro particles) and small EVs (<200 nm, previously nano EVs, including exosomes) (Welsh *et al.*, 2024). Exosomes is a term that was frequently misused to describe all EVs, but this refers specifically to small EVs produced via an endosomal pathway (Witwer and Théry, 2019; Welsh *et al.*, 2024). Large EVs and non-exosomal small EVs are thought to be produced via outward budding of the plasma membrane. Apoptotic bodies are a type of large EVs produced from cells undergoing apoptosis (Théry *et al.*, 2018; Doyle and Wang, 2019; Welsh *et al.*, 2024).

In addition to nomenclature, the MISEV guidelines provide comprehensive recommendations for EV isolation and characterization. Effective isolation requires the separation of EVs from other particles that may be present within the bio-fluid under investigation. No single isolation method is optimal for all samples, and the choice of technique varies depending on the balance between purity, yield, functional integrity, and the end use of the EVs (Clos-Sansalvador *et al.*, 2022). For characterization, the MISEV guidelines recommend assessing EV purity through characterization of proteins that should be found in EV-enriched populations, including markers such as tumour susceptibility gene 101 (TSG101) and tetraspanins (CD9, CD63, CD81) (Welsh *et al.*, 2024). In addition, protein markers indicative of common contaminants should be shown to be absent from EV preparations, and images of individual EVs (e.g. electron micrographs) and an analysis of the biophysical properties, such as the size and concentration of EVs, should be included in any publication of EVs (Welsh *et al.*, 2024).

EVs have been chosen as the overarching term used throughout this review. However, it is important to note that the main techniques used for analysis (e.g. nanoparticle tracking analysis) measure particles that may include non-vesicular particles, such as protein aggregates and lipoproteins (Welsh *et al.*, 2024).

In an IVF setting, it has been demonstrated that EVs are released into the media by embryos during *in vitro* culture, and these EVs are hypothesized to be released within the reproductive tract during the establishment of a natural pregnancy (Bridi *et al*., 2020; Veraguas *et al.*, 2021). In support of this, studies have demonstrated that embryo-derived EVs are taken up by the oviduct or endometrial cells, leading to alterations in gene expression (Dissanayake *et al.*, 2021; Aguilera *et al.*, 2023; Hamdi *et al.*, 2024; Makieva *et al.*, 2024). EVs have also been detected in the embryo culture media of various mammalian species, with research exploring how their characteristics vary in relation to embryo quality. Due to their ability to modify gene expression of endometrial cells, embryo-derived EVs are hypothesized to serve as biomarkers for developmental competence, potentially helping to predict implantation success and live birth outcomes. This potential could make them valuable tools for improving embryo selection in ARTs such as IVF or ICSI.

This systematic review aims to provide a summary of the evidence that embryo-derived EVs are associated with the developmental competence of the embryo. The review also identifies the substantial knowledge gaps in the current literature and provides important suggestions for future work, with the aim of inspiring high-quality quantitative research that progresses this field.

## Methods

### Expectations/hypotheses

We expect to retrieve studies that have isolated and analysed EVs from embryo culture media, examining their presence and characteristics. Additionally, we seek to identify studies that compare EV characteristics between high-quality and low-quality embryos. Quality will be measured in a range of ways, from embryo morphological grading through to clinical pregnancy and live birth.

### Types of studies

Observational studies looking at EVs from the culture media of *in vitro* cultured embryos from mammalian species.

### Types of participants

Embryos from all mammalian species.

### Outcome(s)

The outcomes of this systematic review were (i) characterization of general embryo EV concentration, size, and cargo such as DNA, RNA, and proteins; and (ii) assessment of EV concentration, size, and cargo and their association with embryo morphological quality, pregnancy outcomes, and live birth rates.

### Search strategy and screening

A systematic search of the literature was conducted in five databases, MEDLINE, EMBASE, BIOSIS Previews, Web of Science, and Scopus, to retrieve publications that have studied and characterized embryo-derived EVs from mammalian species. The search was performed according to the Preferred Reporting Items for Systematic Reviews and Meta-Analyses (PRISMA) guidelines (Page *et al.*, 2021), with the protocol registered on Open Science Framework (https://doi.org/10.17605/OSF.IO/TPXVU). The search was conducted, and outputs were retrieved on 14 January 2026, with the search for keywords ‘Embryo’ AND ‘Extracellular Vesicle’; many synonyms were included for each keyword in the search, and appropriate medical subject headings were used for each database. The detailed search strategies are outlined in Supplementary Data File S1. No limitations on publication date or language were used. Records not available in English were translated using automated software or read by a colleague fluent in the language. Additionally, references of included studies were screened to detect any further studies that may have been missed by the search. Authors of abstract-only studies that met our inclusion criteria were contacted to enquire about access to full text results; in addition, Scopus was searched using the authors’ name to determine if the abstract had been subsequently published as a full text record. If no full text was provided and/or no records identified, these abstract-only manuscripts were excluded.

Title and abstract screening was performed by one screener (G.B.), with a second screener (L.C.) screening every 10th record, along with all Yes/Maybe’s from the first screener. Full text screening was performed by two screeners (G.B. and L.C.), with a third screener (L.W.C. or V.J.) consulted for any conflicts or records requiring further discussion. Full text screening was managed within Covidence (Covidence, 2024).

### Eligibility and study selection

Studies were included if they had collected *in vitro* culture media from mammalian embryos and had isolated and assessed EVs from it. Reasons for exclusion were: ‘Non mammalian embryos’, ‘No collection of embryo culture media’, ‘Duplicate of another full text’, ‘No full text available’, ‘No characterization or assessment of EVs’, ‘Not embryo-derived EVs’, and ‘Review/Theory/Lacking primary data’.

The last stage of eligibility was to check the integrity of the included studies by searching the record titles within the Retraction Watch database; any retracted records would be removed from the review (The Center for Scientific Integrity, 2018).

### Data extraction and management

A data extraction template was designed within Covidence, collecting data on sample numbers, media used, culture periods, EV isolation and characterization techniques used, EV concentration, size and cargo reported, and whether embryo EVs were studied from different quality embryos. Data extraction was performed by one reviewer (G.B.). All extracted data were then exported and managed within Excel for curation of results (Microsoft Corporation, 2024). Measurements of error for EV concentration or size reported as CIs, SEM, and interquartile range were transformed to SD of error (Wan *et al.*, 2014; Higgins *et al*., 2024a). Data analyses from the included studies were synthesized using a narrative approach.

### Data synthesis

Data were extracted to address three key outcomes: (i) characterizing embryo-derived EV populations by reporting EV concentration, size, and contents/cargo, (ii) evaluating the association between EV characteristics and embryo quality, and (iii) examining the association between EV characteristics and clinical outcomes. Developmental competence was chosen as the terminology to collectively refer to studies assessing embryo quality morphologically and those assessing clinical outcomes. To synthesize data, vote counting based on direction of effect was used to assess the associations between EV characteristics and developmental competence (McKenzie and Brennan, 2024).

### Risk of bias assessment

Risk of bias (RoB) analysis was performed using a modified version of the Newcastle–Ottawa Scale (NOS) that was adapted to suit the design and context of the included studies (Blanchard *et al.*, 2024) (Supplementary Data File S2). The seven criteria for bias were assessed by one reviewer (G.B.) and reported for all included studies (Supplementary Table S1).

### Certainty assessment/evidence grading

GRADE assessment was performed to establish the certainty in the outcomes being assessed (McMaster University and Evidence Prime, 2023). Study design, RoB, inconsistency of results, representativeness of the samples used, and number of studies found were all used to grade the evidence. This assessment is provided in Supplementary Table S2.

## Results

### Search results

**Figure 1.**
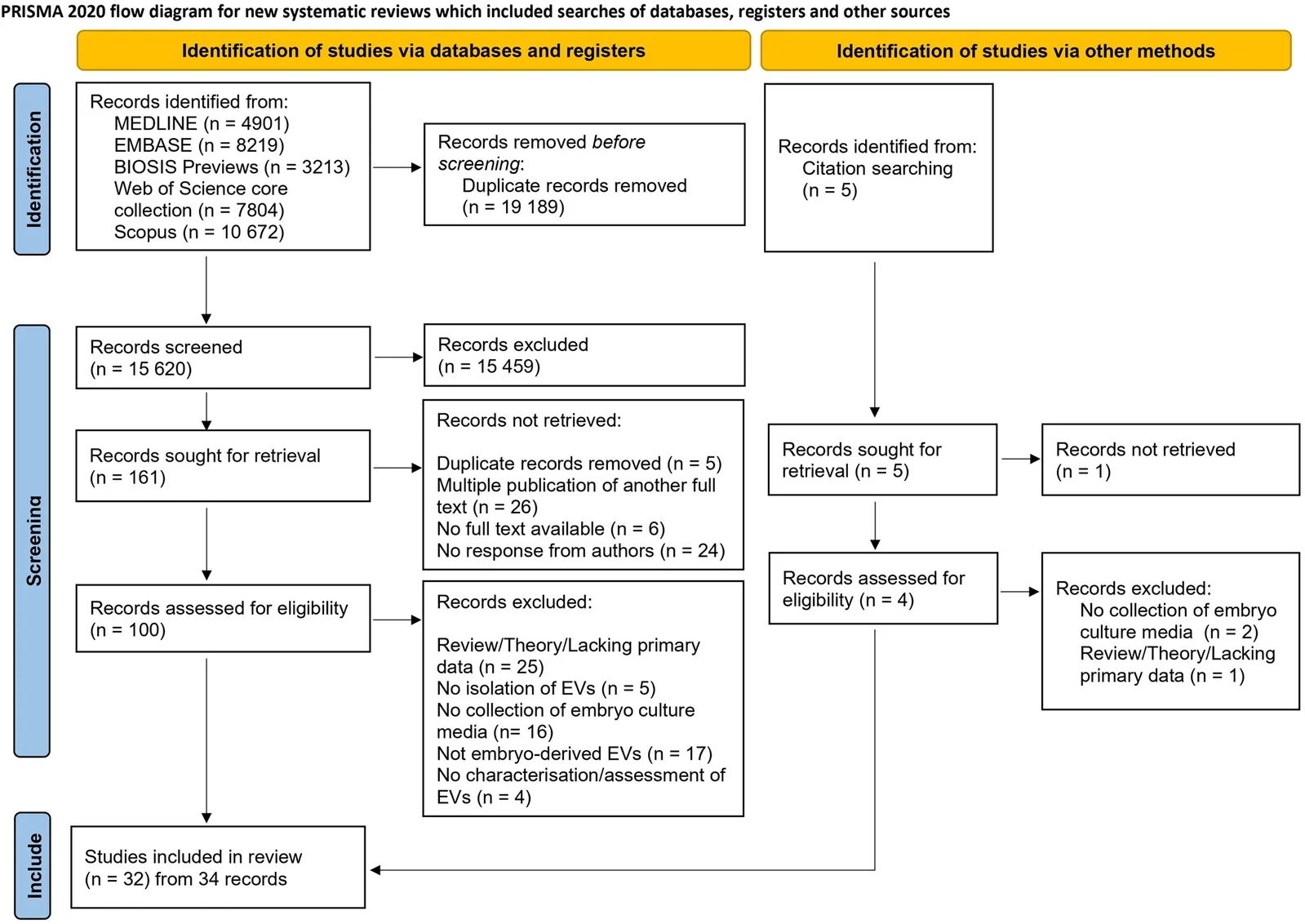
**PRISMA search flow diagram**. EV, extracellular vesicle; PRISMA, Preferred Reporting Items for Systematic Reviews and Meta-Analyses.

The search results are presented in the PRISMA flow diagram (Fig. 1). A total of 34 809 records were retrieved: with 4901 from MEDLINE, 8219 from EMBASE, 3213 from BIOSIS Previews, 7804 from Web of Science, and 10 672 from Scopus. Duplicates were removed, leaving 15 620 records, of which the title and abstracts were screened according to the predetermined inclusion and exclusion criteria. The remaining 161 records were then retrieved and imported into Covidence to manage the full text screening. Five additional duplicates were identified. Authors of 56 ‘abstract only’ records were contacted to enquire if full texts had been published. A response was received from 12 (response rate: 21.4%), with an additional 20 identified as ‘multiple publication of another full text’. These ‘abstract only’ records were excluded if no response was received from the authors, no full text was available, or if the full text provided was a duplicate of a record already identified in this review. This resulted in 100 records screened during the full text screening stage. Sixty-seven records were excluded based on the inclusion and exclusion criteria during the full text screening. All 33 included records had their reference lists screened, which identified one additional, relevant record. A total of 32 studies identified from 34 records were included in this review. Two of the identified records (Bridi, 2021; Fan *et al.*, 2024b) were from the same studies as two other records (Bridi *et al.*, 2021; Pavani *et al.*, 2022). The included studies were further categorized into two groups: (i) studies that characterized general embryo EV samples without comparing embryo developmental competence and (ii) studies that assessed embryo-derived EVs and their association with outcomes, i.e. high versus low morphological quality, aneuploid versus euploid, or pregnancy versus non-pregnancy.

All included studies were checked within the Retraction Watch database, and no concerns or retractions of these studies were found (The Center for Scientific Integrity, 2018).

### Summary of included study characteristics

All included studies isolated and characterized EVs collected from the *in vitro* culture media of embryos (Table 1). Twenty-one of the included studies examined EVs from bovine embryos (Mellisho *et al.*, 2017, 2019; Qu *et al.*, 2017; Pavani *et al.*, 2019, 2020, 2022; Taqi *et al.*, 2019; Dissanayake *et al.*, 2020, 2021; Melo-Báez *et al.*, 2020, 2023; Bridi *et al.*, 2021; Aguilera *et al.*, 2023, 2024; Caamaño *et al.*, 2024; De Bem *et al.*, 2024; Hamdi *et al.*, 2024; Mazzarella *et al.*, 2025a,b; Lange-Consiglio *et al.*, 2026; Townsend *et al.*, 2025), six studies examined EVs from human embryos (Abu-Halima *et al.*, 2017; Giacomini *et al.*, 2017; Marin, 2019; Vyas *et al.*, 2019; Veraguas *et al.*, 2021; Makieva *et al.*, 2024), three studies examined EVs from murine embryos (Kim *et al.*, 2019; Simon *et al.*, 2020; Nagy *et al.*, 2025), one study examined EVs from porcine embryos (Saadeldin *et al.*, 2014), and one study examined EVs from ovine embryos (Burns *et al.*, 2016). IVF/*in vitro* production was the most common method of embryo production used in 21 studies (Table 1). However, ICSI was the dominant method for human embryo studies, performed in five of the six human studies, whilst the remaining study did not state their method of fertilization (Marin, 2019). Four studies (Bridi *et al.*, 2021; Aguilera *et al.*, 2023, 2024; De Bem *et al.*, 2024) examined EVs from *in vivo*-produced bovine embryos. Four studies (Burns *et al.*, 2016; Kim *et al.*, 2019; Simon *et al.*, 2020; Nagy *et al.*, 2025) focused on EVs from murine or ovine embryos produced from natural mating. All embryos produced *in vivo* or by natural mating were subsequently *in vitro* cultured. Two bovine studies and the porcine study (Saadeldin *et al.*, 2014; Mellisho *et al.*, 2017; Caamaño *et al.*, 2024) used parthenogenic activation (PA), and two bovine studies used somatic cell nuclear transfer (Qu *et al.*, 2017; De Bem *et al.*, 2024).

**Table 1.** Summary of included studies.

Species studied	Reference	Method of embryo production	Culture media at EV collection	Group or individual culture at EV collection	Culture time period EVs are from	EV isolation techniques	Supernatant or resuspended pellet analysed	EV characterization techniques	Pooled or individual samples analysed
Human	Abu-Halima *et al.*, 2017	ICSI	CSC Medium (Irvine Scientific)	Individual	Days 1–3 or 1–5	Centrifugation (5000*g*)	Supernatant	NTA, dynamic light scattering, electron microscopy	Individual
	Giacomini *et al.*, 2017	IVF or ICSI	Quinn’s Advantage Cleavage or Blastocyst medium (Cooper Surgical) + 10% SSS (Irvine Scientific)	Group	Days 1–3 or 3–5	Centrifugation (300*g*, 2000*g*, 5000*g*, 110 000*g*)	Resuspended pellet	NTA, TEM, western blot, RNA analysis, HLA-G detection	Group cultured: NTA Pooled: TEM, western blot, RNA analysis, HLA-G detection
	Marin, 2019	Not provided	Blast sequential culture medium (Origio)	Individual or group	Days 3–5	Polymer precipitation (ExoQuick PLUS Exosome Purification Kit) or SEC or centrifugation (300*g*, 2000*g*, 5000*g*, 100 000*g*)	Resuspended pellet and original culture media	NTA, TEM	Individual: NTA, TEM Pooled: TEM of other samples
	Vyas *et al.*, 2019	ICSI	Global media (Life Global)	Individual	Days 1–3 or 1–5/6	Centrifugation (300*g*, 2000*g*, 12 000*g*, 49 000 rpm for TEM; 200*g*, 2000*g*, 14 000*g* for NTA)	Supernatant	TEM and NTA	Pooled
	Veraguas *et al.*, 2021	ICSI	Global Total^®^ (LifeGlobal)	Individual	Days 1–3	Centrifugation (700*g*, 2500*g*)	Supernatant and resuspended pellet	NTA, TEM, flow cytometry, gDNA analysis	Individual
	Makieva *et al.*, 2024	ICSI	Quinn’s Advantage Cleavage or Blastocyst + SSS	Individual	Days 1–3 or 3–5	Days 1–3: Centrifugation (300*g*, 2000*g*, 5000*g*, 110 000*g*)	Resuspended pellet (Days 1–3) and original culture media (Days 3–5)	NTA, TEM, RNA analysis	Individual: NTA of Days 3–5 EVs Pooled: NTA for Days 1–3 EVs, TEM, RNA analysis
Bovine	Mellisho *et al.*, 2017	IVP or PA	SOFdep	Individual or group	Days 7–9	Individual: Centrifugation (800*g*, 2000*g*) Pooled: Centrifugation (800*g*, 2000*g*, 120 000*g*) Group: Centrifugation (10 000*g*, 30 000*g*, 100 000*g*)	Resuspended pellet (group and pooled) and supernatant (individual)	TEM, NTA, flow cytometry	Individual: NTA Pooled: flow cytometry Group cultured: TEM
	Qu *et al.*, 2017	SCNT	Basic culture medium + myo-inositol, ITS, EGF, and PVA	Group	Days 1–3	Centrifugation (300*g*, 2000*g*, 10 000*g*, 100 000*g*)	Resuspended pellet	TEM, Immunofluorescence	Pooled
	Mellisho *et al.*, 2019	IVP	SOFdep	Individual	Days 5–7.5	Centrifugation (700*g*, 2500*g*)	Supernatant	NTA, TEM, flow cytometry, RNA analysis	Individual: NTA, TEM, flow cytometry Pooled: RNA analysis
	Pavani *et al.*, 2019	IVP	Ultracentrifuged SOF + ITS + BSA	Group	Days 1–8	OptiPrep™ Density Gradient, Fractions 8–9 centrifuged (100 000*g*)	Resuspended pellet	NTA, TEM, western blotting	Pooled
	Taqi *et al.*, 2019	IVP	SOF + 10% exosomes-depleted foetal bovine serum	Group	Days 1–8	Differential centrifugation (300*g*, 2000*g*, 10 000*g*, 120 000*g*)	Resuspended pellet	Western blot, NTA, TEM, RNA analysis	Group cultured
	Dissanayake *et al.*, 2020	IVP	SOFaaci containing 0.8% BSA	Individual	Days 1–2, 1–5, or 1–8	Centrifugation (400*g*, 2000*g*) then SEC (Fractions 6–9)	SEC fractions	NTA, EV array, TEM, SEM	Individual: NTA Pooled: EV array, TEM, SEM
	Melo-Báez *et al.*, 2020	IVP	SOFdep	Individual	Days 3.5–5	Individual: Centrifugation (700*g*, 2000*g*) Pooled: Centrifugation (700*g*, 2000*g*, 10 000*g*, 100 000*g*)	Supernatant	NTA, TEM, flow cytometry, RNA analysis	Individual: NTA Pooled: TEM, flow cytometry, RNA analysis
	Pavani *et al.*, 2020	IVP	Ultracentrifuged SOF + ITS + BSA	Group	Days 1–8	Centrifugation (10 000*g*, 30 000*g* 100 000*g*) or OptiPrep™ Density Gradient or SEC	Resuspended pellet or SEC fractions	Western blot, TEM, NTA	Pooled
	Bridi *et al.*, 2021	IVV or IVP	SOFaaci + BSA, sodium pyruvate, gentamicin	Individual	Days 7–9	Centrifugation (16 500*g*), Exoquick-TC, centrifugation (16 500*g*, 120 000*g*)	Resuspended pellet	TEM, NTA, RNA analysis	Pooled
	Dissanayake *et al.*, 2021	IVP	SOFaaci containing 0.8% BSA	Individual	Days 1–5	Centrifugation (400*g*, 2000*g*), SEC (Fractions 6–9 used)	SEC fractions	NTA	Pooled
	Pavani *et al.*, 2022	IVP	SOFaa + ITS + BSA	Individual	Days 1–8	SEC (Fractions 6–15 used)	SEC fractions	NTA, TEM, western blotting, RNA analysis	Pooled
	Aguilera *et al.*, 2023	IVV or IVP	SOFdep	Individual	Days 5–7	Centrifugation (400*g*, 2000*g*) then ExoLutE^®^ Conditioned Medium exosome isolation kit	Resuspended from EV enrichment procedure	NTA, flow cytometry, TEM	Pooled
	Melo-Báez *et al.*, 2023	IVP	SOFdep	Individual	Days 3.5–5 or 5–7	Centrifugation (700*g*, 2000*g*)	Supernatant	NTA, TEM, flow cytometry, RNA analysis	Individual: NTA Pooled: TEM, flow cytometry, RNA analysis
	Aguilera *et al.*, 2024	IVV or IVP	SOFdep	Individual	Days 7–9	Centrifugation (400*g*, 2000*g*) then ExoLutE^®^ Conditioned medium exosome isolation kit	Resuspended from EV enrichment procedure	NTA, flow cytometry, TEM	Pooled
	Caamaño *et al.*, 2024	IVP and PA	SOFdep	Individual	Days 5–7	Pooled: centrifugation (700*g*, 2000*g*, 10 000*g*, 4000*g* for concentration)	Supernatant	NTA, western blot, TEM, DNA analysis	Individual: NTA, DNA analysis Pooled: NTA, western blot, TEM
	De Bem *et al.*, 2024	IVV, IVP, and cloned blastocyst (SCNT)	SOF + FBS, BSA, pyruvate and gentamycin	Individual	6 h on Day 16	Centrifugation (300*g*, 2000*g*, 16 500*g*, 100 000*g*)	Resuspended pellet	NTA, TEM, western Blot, RNA analysis	Individual
	Hamdi *et al.*, 2024	IVP	SOFaa + 5% EV-depleted foetal calf serum	Group	53–77 h (∼Days 3–4)	Centrifugation (300*g*, 2000*g*, 12 000*g*), SEC, centrifugation (100 000*g*)	Resuspended pellet	TEM, NTA, flow cytometry, RNA analysis	Group: NTA, flow cytometry Pooled: TEM, RNA analysis
	Mazzarella *et al.*, 2025a	IVP	SOF	Group	6 h on Day 7	Centrifugation (300*g*, 10 000*g*), SEC (PURE-EV^®^ columns)	SEC fractions	NTA, TEM, flow cytometry, protein analysis	Group cultured
	Mazzarella *et al.*, 2025b	IVP	SOF	Group	6 h on Day 3.5	Centrifugation (300*g*, 10 000*g*), SEC (PURE-EV^®^ columns)	SEC fractions	NTA, TEM, flow cytometry, protein analysis	Group cultured
	Townsend *et al.*, 2025	IVP	CR1-aa medium	Individual	Days 5–8	Centrifugation (3000*g*), miRCURY Exosome Cell/Urine/CSF Kit	Resuspended from EV enrichment procedure	NTA, RNA analysis	Pooled
	Lange-Consiglio *et al*., 2026	IVP	IVC medium (Stroebech)	Group	Days 5–7 and 7–11	Centrifugation (250*g*, 4000*g*, 100 000*g*)	Resuspended pellet	NTA, western blot, TEM	Group cultured
Murine/mice	Kim *et al.*, 2019	Natural mating	DMEM (Gibco/Life Technologies)	Group	Days 4.5–7.5	Centrifugation (300*g*, 4000*g*, 10 000*g*, 200 000*g*)	Resuspended pellet	TEM, western blot	Pooled
	Simon *et al.*, 2020	Natural mating	Oxygenated G2-Plus media (Vitrolife)	Group	Days 1.5–4.5	Centrifugation (300*g*, 2000*g*—pelleted ABs, 185 000*g*—pelleted non-apoptotic EVs)	Resuspended pellet	TEM, immunogold, NTA, DNA sequencing	Pooled
	Nagy *et al.*, 2025	Natural mating	M2 medium (Millipore)	Group	Days 2.5–3.5	exoEasy Midi Kit	Resuspended from EV enrichment procedure	RNA analysis	Pooled
Porcine/Pigs	Saadeldin *et al.*, 2014	PA	PZM-5 (Funakoshi Co)	Group	Days 1–2, 3–4, 5–6, 6–7	Centrifugation (300*g*, 2000*g*, 10 000*g*, 200 000*g*)	Resuspended pellet	TEM, immunofluorescence, RNA analysis	Pooled
Ovine/Sheep	Burns *et al.*, 2016	Natural mating	DMEM/F-12 + 10% exosome-depleted FBS, glutamine, insulin, pyruvate, non-essential amino acids, and penicillin, and streptomycin	Individual	Days 14–15	ExoQuick-TC, centrifugation (15 000*g*)	Resuspended pellet	NTA, mass spectrometry, RNA sequencing	Individual

ABs, apoptotic bodies; BSA, bovine serum albumin; CSC, continuous single culture; CR1-aa, Charles Rosenkrans medium with amino acids; DMEM, Dulbecco’s modified Eagle’s media; EGF, epidermal growth factor; EV, extracellular vesicle; FBS, foetal bovine serum; gDNA, genomic DNA; HLA-G, human leukocyte antigen G; ITS, insulin, transferrin, selenium; IVP, *in vitro* production; IVV, *in vivo* production; NTA, nanoparticle tracking analysis; PA, parthenogenetic activation; PVA, polyvinyl alcohol; PZM-5, serum-free chemically defined porcine zygote medium-5; SCNT, somatic cell nuclear transfer; SEC, size exclusion chromatography; SEM, scanning electron microscopy; SOF, synthetic oviduct fluid; SOFaa, SOF enriched with non-essential and essential amino acids; SOFaaci, modified SOF with amino acids, sodium citrate, and myo-inositol; SOFdep, EVs-depleted SOF medium; SSS, serum substitute supplement; TEM, transmission electron microscopy.

Twenty-four of the studies identified and characterized general embryo EV populations (Table 2). Five of these were human studies, of which three assessed EVs from embryos that had reached the cleavage or blastocyst stage of development (Giacomini *et al.*, 2017; Marin, 2019; Makieva *et al.*, 2024), one assessed all embryos regardless of quality (Veraguas *et al.*, 2021), and the remaining study assessed only embryos that arrested during development or had low cell numbers at the blastocyst stage (Vyas *et al*., 2019). Similarly, 14 studies assessed general embryo EV populations in bovines, of which 10 assessed EVs from embryos that reached 8–16 cell, morula, blastocyst, or Day 16 of embryo development (Bridi *et al.*, 2021; Aguilera *et al.*, 2023, 2024; Melo-Báez *et al.*, 2023; Caamaño *et al.*, 2024; De Bem *et al.*, 2024; Mazzarella *et al.*, 2025a,b; Townsend *et al.*, 2025; Lange-Consiglio *et al.*, 2026), and the other four studies assessed all embryos regardless of quality (Qu *et al.*, 2017; Pavani *et al.*, 2019, 2020; Taqi *et al.*, 2019). The remaining four studies were performed in other mammalian embryos, of which the ovine study (Burns *et al*., 2016) assessed embryos that reached Day 14 of development and two murine studies (Kim *et al.*, 2019; Nagy *et al.*, 2025) assessed embryos that reached either the blastocyst or two-cell stage, respectively, whilst the porcine study (Saadeldin *et al.*, 2014) and the final murine study (Simon *et al.*, 2020) assessed all embryos regardless of embryo quality.

**Table 2.** Embryo EV characterization—irrespective of developmental competence.

Species	Reference	Quality of embryos included	Concentration of EVs reported (EVs/ml)	Concentration of EVs in control (EVs/ml)	Size of EVs reported (nm)		Mean size of EVs in control (nm)	Cargo of EVs reported	Cargo of control
					Mean size	Mode size			
Human	Giacomini *et al.*, 2017	Cleavage or expanded blastocyst (zero degeneration)	Days 1–3: 1.2 × 10^12^^!^ Days 3–5: 1.6 × 10^12^^!^	Culture media only: ∼0 Culture media + SSS: 6.00 × 10^11^^!^	D3-EVs: 80.0^!^ D5-EVs: 78.0^!^	–	Culture media only: ∼0 SSS-EVs: 71.0^!^	*POU5F1* and *NANOG* mRNA detected in D3-EVs and D5-EVs *HLA-G* detected in D5-EVs primarily	No detectable levels of transcripts or HLA-G
	Marin, 2019	Blastocysts at Day 5	Days 3–5: 8.86 × 10^8^	4.28 × 10^8^	SEC: 57.05	–	SEC: 44.47	–	–
	Vyas *et al.*, 2019	All unsuitable for infertility treatment use	Days 1–3: 7.0 × 10^8^ Days 1–5/6: 7.89 × 10^8^	Unconditioned media: 7.33 × 10^8^	–	–	–	–	–
	Veraguas *et al.*, 2021	TOP, FAIR and POOR quality, and arrested embryos	ABs: 5.34 × 10^8^ MVs/Exo: 12.98 × 10^8^	–	ABs: 118.08 MVs/Exo: 108.45	ABs: 94.19 MVs/Exo: 90.12	–	–	–
	Makieva *et al.*, 2024	Embryos and blastocyst (zero degeneration)	D3 EV: 2.62 × 10^10^	–	D3 EV: 191.1	–	–	76% protein coding, 13% long non-coding RNA 17.9% RNA overlap in pooled replicates	Negligible level of transcripts in control culture media
Bovine	Qu *et al.*, 2017	All	–	–		90 < exosomes ≤ 120	–	–	–
	Pavani *et al.*, 2019	All	2 ml SCM: 236.5 × 10^8^ 1 ml SCM: 40.8 × 10^8^	Blank BSA medium: 53.7 × 10^7^	2 ml SCM: 123.7 1 ml SCM: 133.8	2 ml SCM: 92.8 1 ml SCM: 97.5	Blank BSA medium: 149.1	–	–
	Taqi *et al.*, 2019	All	5% O_2_: 1.48 × 10^8^ (male) 1.75 × 10^8^ (female) 20% O_2_: 2.15 × 10^8^ (male) 1.37 × 10^8^ (female)	–	5% O_2_: ∼115 (male) ∼115 (female) 20% O_2_: ∼120 (male) ∼107 (female)	–	–	Varying levels of *NFE2L2*, *SOD1*, and *NOTCH1* detected between the groups	–
	Pavani *et al.*, 2020	All	DU: 3.02 × 10^10^ ODG: 5.50 × 10^9^ SEC: 2.09 × 10^10^, 1.50 × 10^11^, and 1.13 × 10^10^	–	DU: 266.8 ODG: 151.3 SEC: 119.2	DU: 186.2 ODG: 100.1 SEC: 99.7	–	–	–
	Bridi *et al.*, 2021	Hatched blastocysts	*In vivo*: 1.63 × 10^9^ *In vitro*: 3.62 × 10^9^	–	*In vivo*: 130.90 *In vitro*: 137.95	–	–	112 miRNAs identified in total 3 and 5 miRNA exclusive to *in vivo* and *in vitro* EVs, respectively 14 miRNAs upregulated and 2 miRNA downregulated for *in vivo* vs *in vitro*	–
	Aguilera *et al.*, 2023	Grades 1 and 2 expanded blastocysts^1^	*In vivo*: 4.24 × 10^9^ *In vitro*: 1.14 × 10^9^	–	*In vivo*: 186.4 *In vitro*: 168.9	*In vivo*: 149.2 *In vitro*: 133.7	–	–	–
	Melo-Báez *et al.*, 2023	Morula and blastocysts	Compaction: 9.4 × 10^8^ Blastulation: 2.4 × 10^9^	Minimal	Compaction: 109.4 Blastulation: 106.6	–	–	140 miRNA identified in total 11 and 47 miRNAs exclusive to compaction and blastulation EVs, respectively 17 miRNA upregulated and 4 miRNA downregulated in compaction vs blastulation	–
	Aguilera *et al.*, 2024	Quality 1 and 2 blastocysts^1^	*In vivo*: 2.12 × 10^10^ *In vitro*: 1.18 × 10^10^	–	*In vivo*: 180.5 *In vitro*: 179.7	*In vivo*: 138.7 *In vitro*: 135.4	–	–	–
	Caamaño *et al.*, 2024	Blastocyst and arrested morula	8.84 × 10^9^	–	147.62	–	–	–	–
	De Bem *et al.*, 2024	Appropriately developed conceptuses	*In vivo*: 1.11 × 10^9^ *In vitro*: 2.14 × 10^9^ Clones: 2.69 × 10^9^	–	*In vivo*: 155.35 *In vitro*: 159.14 Clones: 171.2	–	–	359, 321, and 384 miRNAs identified in *in vivo*, *in vitro*, and cloned conceptuses EVs, respectively. 4 miRNA upregulated and 2 miRNA downregulated in *in vivo* vs *in vitro*. 3 miRNA upregulated and 5 miRNA downregulated in *in vivo* vs cloned.	–
	Mazzarella *et al.*, 2025a	Excellent or good quality at Day 7	2.84 × 10^8^	PBS—zero particles	159	–	PBS—zero particles	82 proteins identified.	–
	Mazzarella *et al.*, 2025b	8–16 cell stage	2.08 × 10^8^	PBS—zero particles	163	–	PBS—zero particles	111 proteins identified.	–
	Townsend *et al.*, 2025	Morula^1^	–	–	285.8	–	–	3 full length mRNA transcripts (SLBP2, ACCSL, DPPA3) identified in XX and XY embryo.	No amplification of transcripts in control culture media.
	Lange-Consiglio *et al.*, 2026	Morula	Days 5–7: 1.5 × 10^11^^!^ Days 7–11: 1.8 × 10^11^^!^	–	Days 5–7: 230^!^ Days 7–11: 210^!^	–	–	–	–
Murine/mice	Kim *et al.*, 2019	Blastocysts at 4.5 dpc	–	–	87.1	–	–	–	–
	Simon *et al.*, 2020	All	naEV: 1.74 × 10^7^#	–	–	–	–	14 genes enriched for ABs vs blank media 8 genes enriched for naEV vs blank media	–
	Nagy *et al.*, 2025	2-cell embryos	–	–	–	–	–	160, 99, and 83 miRNA in control, white light and red light exposed groups, respectively. 13 and 5 miRNA exclusive to white and red light embryo EVs. 30 miRNA downregulated and 24 upregulated in the white light group vs control. 38 miRNA downregulated and 24 upregulated in red light group vs control.	–
Porcine/Pigs	Saadeldin *et al.*, 2014	All	–	–	–	–	–	*Oct4*, *Sox2*, *Klf4* expressed in all samples *c-MYC* found only in PPA at Day 2 and PA and PPA at Day 6 *NANOG* only expressed later in development (Day 7)	Positive control contained mRNA for all genes Negative controls were negative for all mRNA
Ovine/Sheep	Burns *et al.*, 2016	Intact Day 14 conceptuses	5.8 × 10^8^$	–	150	–	–	231 proteins identified 512 mRNAs detected	–

ABs, apoptotic bodies; BSA, bovine serum albumin; dpc, days post conception; DU, differential ultracentrifugation; EV, extracellular vesicle; Exo, exosomes; HLA-G, human leukocyte antigen G; miRNA, microRNA; MVs, microvesicles; naEV, non-apoptotic EV; ODG, OptiPrep™ density gradient; PA, parthenogenetic activation; PPA, punctured parthenogenetic activation; SCM, spent culture medium; SEC, size exclusion chromatography; SSS, serum substitute supplement.

# Net EV concentration after subtracting EVs in the same volume of blank media.

1 IETS grading scheme used.

$ Calculated from culture volume and total particle count.

! Estimated off a graph (exact values not provided).

**Figure 2.**
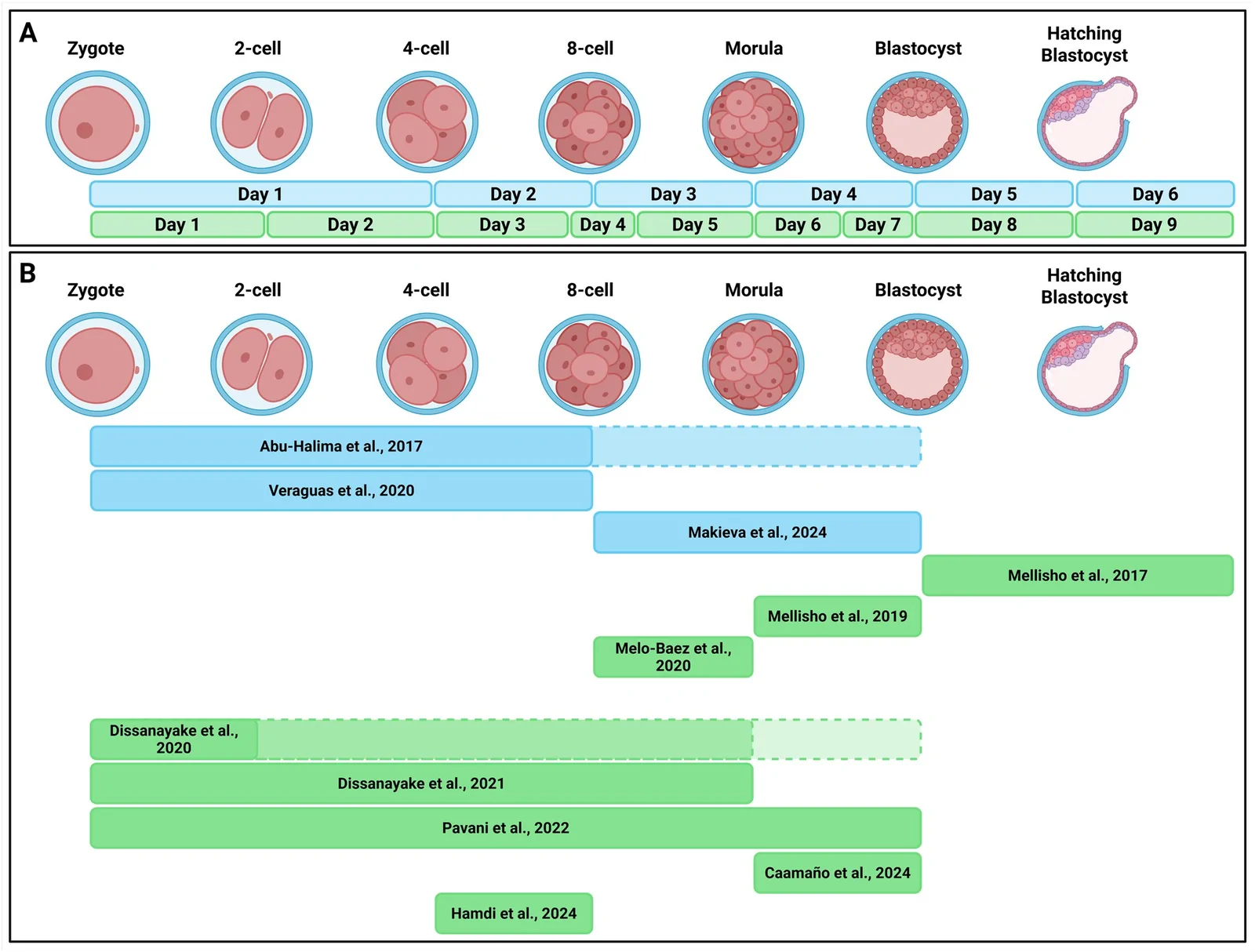
**Developmental trajectory of human vs bovine embryos (A) and time period of EVs analysed for association with developmental competence (B).** (A) Represents the variation in days of development between human (blue) and bovine (green). (B) Represents the developmental periods that EVs were collected from in studies assessing the association between embryo EVs and developmental competence (Tables 3 and 4). Dashed lines indicate studies that assessed embryo EVs from multiple different timepoints (i.e. Dissanayake, 2020, studied embryo EVs from Days 1–2, 1–5, and 1–8 of culture). Human embryo studies are indicated in blue, whilst bovine embryo studies are indicated in green. Created in BioRender. Boom (2026) https://BioRender.com/v30e071. EV, extracellular vesicle.

Eleven studies included in this review characterized embryo-derived EVs and assessed the association between EV concentration and size and developmental competence (Table 3). Seven of these papers, five bovine and two human, also assessed the association between EV cargo and embryo developmental competence (Table 4) (Mellisho *et al.*, 2019; Melo-Báez *et al.*, 2020; Veraguas *et al.*, 2021; Pavani *et al.*, 2022; Caamaño *et al.*, 2024; Hamdi *et al.*, 2024; Makieva *et al.*, 2024). To aid data interpretation, Fig. 2 represents both the variation in developmental timepoints between human and bovine (Fig. 2A) and the vast range of timepoints that EVs were sampled from across the two species for studies assessing the association of EV characteristics with developmental competence (Fig. 2B).

**Table 3.** Embryo EV size and concentration—associated with developmental competence.

Species studied	Reference	Quality assessment: timepoint and method	Time of EV sampling	Sampling information	Mean concentration of EVs reported (EVs/ml) ± SD	Direction of association of EV concentration with good developmental competence	Size of EVs reported (nm) ± SD		Direction of association of EV size with good developmental competence
							Mean	Mode	
Human	Abu-Halima *et al.*, 2017	Positive pregnancy detection	Days 1–3 and 1–5	Individual (EV concentration n = 8 total; EV size n = 2 total)	Positive pregnancy: 3.8 × 10^9^ Negative pregnancy: 7.35 × 10^9^	↓	–	Positive outcome: 148.8 Negative outcome: 141.7	↑
	Veraguas *et al.*, 2021	Day 3—Blastomere number and morphological quality	Days 1–3	Individual (number not specified)	MVs/Exo: TOP: 14.26 ± 18.0^!^ × 10^8^ FAIR: 11.85 ± 4.5^!^ × 10^8^ POOR: 12.94 ± 11.5^!^ × 10^8^ ABs: TOP: 3.26 ± 3.7^!^× 10^8^ FAIR: 7.54 ± 22.0^!^ × 10^8^ POOR: 2.79 ± 1.0^!^ × 10^8^	↔	MVs/Exo: TOP: 112.17 ± 14.5^!^ FAIR: 108.02 ± 8.0^!^ POOR: 102.78 ± 9.0^!^ ABs: TOP: 118.64 ± 16.5^!^ FAIR: 118.41 ± 11.5^!^ POOR: 114.54 ± 10.5^!^	MVs/Exo: TOP: 91.74 ± 8.0^!^ FAIR: 89.67 ± 5.0^!^ POOR: 88.17 ± 6.5^!^ ABs: TOP: 93.91 ± 9.0^!^ FAIR: 94.68 ± 10.0^!^ POOR: 93.28 ± 6.5^!^	↑
	Makieva *et al.*, 2024	Day 5/6/7—PGT-A biopsy	D5 EVs: Days 3–5	Individual (n = 5 per group)	D5 EVe: 4.89 ± 0.54 × 10^10^$ D5 EVa: 3.36 ± 0.37 × 10^10^$	↑	D5 EVe: 148.1 ± 32.42 D5 EVa: 156.6 ± 52.87	–	↔
Bovine	Mellisho *et al.*, 2017	Day 11—embryo growth performance and diameter	Days 7–9	Individual (n = 10 per group)	IVF-CB: 6.7 ± 3.1 × 10^8^ PA-CB: 4.7 ± 1.3 × 10^8^ IVF-NCB: 8.5 ± 3.7 × 10^8^ PA-NCB: 3.9 ± 1.2 × 10^8^	↔	IVF-CB: 107.3 ± 12.9 PA-CB: 107.0 ± 13.3 IVF-NCB: 122.0 ± 28.7 PA-NCB: 104.5 ± 18.4	IVF-CB: 75.1 ± 7.3 PA-CB: 83.4 ± 10.6 IVF-NCB: 78.9 ± 13.5 PA-NCB: 81.3 ± 16.2	↔
	Mellisho *et al.*, 2019	Day 11—embryo quality, developmental stage and diameter	Days 5–7.5	Individual (V-EB: n = 73 V-LB: n = 61 NV-EB: n = 68 NV-LB: n = 52)	V-EB: 21.40 ± 9.38 × 10^8^ V-LB: 24.90 ± 9.71 × 10^8^ NV-EB: 57.45 ± 20.23 × 10^8^ NV-LB: 21.70 ± 9.50 × 10^8^	↔	V-EB: 122.90 ± 14.36 V-LB: 86.67 ± 9.92 NV-EB: 80.59 ± 21.34 NV-LB: 89.79 ± 11.26	V-EB: 74.15 ± 9.37 V-LB: 70.00 ± 10.19 NV-EB: 65.57 ± 9.14 NV-LB: 70.87 ± 12.91	↔
	Dissanayake *et al.*, 2020	Day 8—embryonic development based on morphological parameters	Days 0–2, 0–5, or 0–8.	Individual Day 2 (good quality n = 23, bad quality n = 22) Day 5 (good quality n = 25, bad quality n = 19) Day 8 (good quality n = 24, bad quality n = 20)	Day 2 good quality: 5.86 ± 1.73^!@^ × 10^8^ Day 2 bad quality: 8.25 ± 4.67^!@^ × 10^8^ Day 5 good quality: 6.40^!^ ± 1.94^!@^ × 10^8^ Day 5 bad quality: 6.20^!^ ± 1.45^!@^ × 10^8^ Day 8 good quality: 5.68 ± 1.18^!@^ × 10^8^ Day 8 bad quality: 7.17 ± 2.14^!@^ × 10^8^	↓	Day 2 good quality: 166 ± 13.9^!@^ Day 2 bad quality: 159 ± 11.3^!@^ Day 5 good quality: 163^!^ ± 12.1^!@^ Day 5 bad quality: 162^!^ ± 12.5^!@^ Day 8 good quality: 163 ± 9.5^!@^ Day 8 bad quality: 154 ± 8.6^!@^	–	↑
	Melo-Báez *et al.*, 2020	Day 7—IETS criteria	Days 3.5–5	Individual (blastocyst: n = 154, arrested: n = 250)	Blastocyst: 9.4 × 10^8^ Arrested: 1.5 × 10^9^	↓	Blastocyst: 109.4 Arrested: 129.6	Blastocyst: 89.1 Arrested: 108.3	↓
	Dissanayake *et al.*, 2021	Day 8—morphology^1^	Days 0–5	Pooled (n = 1, pooled n = 40)	Good quality: 1.598 × 10^10^# Degenerating: 1.296 × 10^10^#	↔	–	–	–
	Pavani *et al.*, 2022	Day 8—morphology	Days 0–8	Pooled (n = 3, pooled n = 26)	Blastocyst: 94.5 ± 1.7 × 10^8^ Arrested: 76.5 ± 8.2 × 10^8^	↑	Blastocyst: 147.4 ± 16.4 Arrested: 118.3 ± 6.1	Blastocyst: 126.5 ± 8.5 Arrested: 98.7 ± 13.2	↑
	Caamaño *et al.*, 2024	Day 7—Blastocyst or arrested morula	Days 5–7	Individual Blast-IVF: n = 22 Blast-PA: n = 8 Arrested morula-IVF: n = 18 Arrested morula-PA: n = 18	Blast-IVF: 6.5 ± 6.1^@^ × 10^9^ Blast-PA: 11.1 ± 14.0^@^ × 10^9^ Arrested morula-IVF: 6.0 ± 5.5^@^ × 10^9^ Arrested morula-PA: 12.0 ± 7.7^@^ × 10^9^	↔	Blast-IVF: 154.5 ± 28.8^@^ Blast-PA: 141.0 ± 21.4^@^ Arrested morula-IVF: 150.6 ± 33.9^@^ Arrested morula-PA: 143.0 ± 45.0^@^	–	↔
	Hamdi *et al.*, 2024	53 h pi—cell number, morphology	Day ∼2–3	Group/pooled (n = 2, n = 25 per group)	Good quality: 1.0^!^ ± 0.1^!^ × 10^9^ Poor quality: 9.8^!^ ± 3.0^!^ × 10^8^	↔	Good-quality embryo: 170^!^ ± 0^!^ Poor-quality embryo: 168^!^ ± 6^!^	Good-quality embryos: 138^!^ ± 10^!^ Poor-quality embryos: 128^!^ ± 17^!^	↑

ABs, apoptotic bodies; CB, competent blastocyst; EB, early blastulation; EV, extracellular vesicle; EVa, EVs from aneuploid embryos; EVe, EVs from euploid embryos; Exo, exosomes; IETS, International Embryo Technology Society; LB, late blastulation; MVs, microvesicles; NCB, non-competent blastocyst; NV, non-viable; PA, parthenogenetic activation; pi, post insemination; V, viable.

Error is reported as SD—except Hamdi *et al*. (2024), which did not state how error was calculated.

# Calculated from total number of EVs loaded onto cells (50 µl of each one).

! Estimated off a graph (exact values not provided).

1 IETS manual used.

@ Error transformed from IQR or 95% CI to SD.

$ Reporting figure caption values, due to discrepancy in values reported.

**Table 4.** Embryo EV cargo—associated with developmental competence.

Species studied	Reference	Quality assessment: timepoint and method	Time period EVs are from	Number of samples analysed for cargo	Cargo of EVs reported
Human	Veraguas *et al.*, 2021	Day 3—blastomere number and morphological quality	Days 1–3	TOP quality: n = 14 ABs/MVs/Exo n = 12 MVs/Exo Arrested: n = 7 ABs/MVs/Exo	Chromosome abnormalities detected in 50–66.6% of TOP quality embryos EVs; and 57.1% of arrested embryo EVs Higher number of chromosome abnormalities in EV gDNA (24.9%) from arrested embryos versus their matched embryos (8.7%)
	Makieva *et al.*, 2024	Day 5/6/7—PGT-A biopsy	D3 EVs: Days 1–3	D3 EVe: n = 5, pooled n = 35 D3 EVa: n = 4, pooled n = 35	66 RNA fragments more abundant in aneuploid vs euploid EVs 129 RNA fragments less abundant in aneuploid vs euploid EVs 4 candidate biomarker RNAs identified (*PPM1J*, *LINC00561*, *ANKRD34C*, *TMED10*)
Bovine	Mellisho *et al.*, 2019	Day 11—embryo quality, developmental stage and diameter	Days 5–7.5	V-EB: n = 3, pooled n = ∼20 V-LB: n = 3, pooled n = ∼20 NV-EB: n = 3, pooled n = ∼20 NV-LB n = 3, pooled n = ∼20	Total of 182 miRNA and 32 snoRNA identified 12 miRNA upregulated and 15 miRNA downregulated in EVs of viable vs non-viable embryos 1 snoRNA unregulated in EVs of viable vs non-viable embryos 8 miRNA exclusive to viable embryos and 24 miRNA exclusive to non-viable embryos 2 snoRNAs exclusive to viable embryo EVs and 3 snoRNAs exclusive to non-viable embryo EVs
	Melo-Báez *et al.*, 2020	Day 7—IETS criteria	Days 3.5–5	Blastocyst: n = 3, pooled n = 10 Arrested: n = 3, pooled n = 10	A total of 95 miRNAs expressed across the samples 4 miRNAs upregulated and 4 downregulated in EVs from embryos that arrested
	Pavani *et al.*, 2022	Day 8 pi—development to blastocyst stage or not	Days 0–8	Blastocyst EVs: n = 3, pooled n = 110 Non-blastocyst EVs: n = 2, pooled n = 110	590 miRNA detected in total 69 differentially expressed miRNA between blastocyst and non-blastocyst EVs 126 tsRNA upregulated and 22 tsRNA downregulated in EVs from blastocyst vs non-blastocyst
	Caamaño *et al.*, 2024	Day 7—blastocyst or arrested morula	Days 5–7	Blast-IVF: n = 22 Blast-PA: n = 8 Arrested morula-IVF: n = 18 Arrested morula-PA: n = 18	Mean DNA concentrations: Blast-IVF: 73.50 ng/µl Blast-PA: 50.66 ng/µl Arrested morula-IVF: 62.34 ng/µl Arrested morula-PA: 108.27 ng/µl
	Hamdi *et al.*, 2024	53hrs pi—Cell number, morphology	Days ∼2–3	GE: n = 2, pooled two groups of n = 25 embryos PE: n = 2, pooled two groups of n = 25 embryos	82 miRNA identified in GE or PE EVs 2 upregulated in GE vs PE EVs 1 exclusive to PE EVs

EB, early blastulation; EV, extracellular vesicle; EVa, EVs from aneuploid embryos; EVe, EVs from euploid embryos; gDNA, genomic DNA; GE, good-quality embryo; IETS, International Embryo Technology Society; LB, late blastulation; miRNA, microRNA; NV, non-viable; PA, parthenogenetic activation; PE, poor-quality embryos; pi, post insemination; snoRNA, small nucleolar RNA; tsRNA, transfer RNA derived-small RNAs, V, viable.

Studies that did not perform appropriate isolation and/or characterization of EVs were excluded from this review (Pallinger *et al.*, 2017, 2018). However, some studies analysing unprocessed spent culture media (SCM) were included, as they had performed appropriate EV isolation on the subsets of EVs used for general characterization and treatment of endometrial cells, thereby meeting the review’s inclusion criteria (Makieva *et al.*, 2024). Additionally, records assessing EVs from the media of co-culture systems, such as embryo–cumulus co-culture or embryo–endometrial cell co-culture, were excluded (Parks *et al.*, 2018; Andrade *et al.*, 2019; Bridi *et al.*, 2025), as the contribution of the embryo-specific EVs would be unclear. Records that assessed EVs in blastocoel fluid were also excluded (Battaglia *et al.*, 2019) due to one of the predetermined inclusion criteria being ‘collection of culture media from embryos’.

### Risk of bias

According to the RoB assessment, four of the six included human studies were low quality, whilst the remaining two were moderate quality (Supplementary Data File S2; Supplementary Table S1). Likewise, 7 of the bovine studies were low quality, and the remaining 14 were moderate quality. The murine, ovine, and porcine studies were all low quality. Small sample sizes, lack of non-respondents’ data, data unadjusted for any confounders/risk factors, and incomplete statistical test information were the main contributors to studies being of lower quality. Of the included studies, only five bovine studies met the gold standard for the RoB criterion ‘ascertainment of the exposure’, defined as complete fulfilment of the MISEV guidelines for EV characterization (Pavani *et al.*, 2019, 2022; Dissanayake *et al.*, 2020, 2021; Caamaño *et al.*, 2024). Based on EV isolation and characterization practices, no human embryo-derived EV study was considered as high quality.

### Outcomes

#### Overview of embryo-derived EVs concentration and size

Twenty-seven of the 32 included studies (84.4%) reported the EV concentration in the embryo SCM; similarly, 27 studies (84.4%) reported a mean and/or mode size of EVs (Tables 2 and 3).

The reported concentrations ranged from 2.79 × 10^8^ EVs/ml to 1.6 × 10^12^ EVs/ml (Giacomini *et al.*, 2017; Veraguas *et al.*, 2021) for the human studies, and 1.37 × 10^8^ EVs/ml to 1.8 × 10^11^ EVs/ml (Taqi *et al.*, 2019; Lange-Consiglio *et al.*, 2026) in the bovine studies. There was wide variation in EV concentrations from media-only controls, for both human and bovine studies, with some reporting very few or no detected particles (Abu-Halima *et al.*, 2017; Mellisho *et al.*, 2019) and others reporting as high as 6.00 × 10^11^ particles/ml (Giacomini *et al.*, 2017). Only one of the murine studies reported an adjusted concentration of EVs in the SCM (1.74 × 10^7^ EVs/ml) by subtracting the concentration of particles in the blank media (Simon *et al.*, 2020). No concentrations were reported for the porcine or ovine studies.

Among the human studies, mean EV size ranged between 57.05 and 191.1 nm (Marin, 2019; Makieva *et al.*, 2024), whilst bovine studies reported a range of 80.59 and 285.8 nm (Mellisho *et al.*, 2019; Townsend *et al.*, 2025). Only one murine study reported a mean EV size of 87.1 nm (Kim *et al.*, 2019), whilst the ovine study reported a mean size of 150 nm (Burns *et al*., 2016). No EV sizes were reported in the porcine study (Saadeldin *et al.*, 2014).

Except for two studies (Pavani *et al.*, 2020; Townsend *et al.*, 2025), all other studies reported EV mean or mode sizes below 200 nm. Therefore, the literature and this review is focused on ‘small EVs’ as defined by the MISEV guidelines (Welsh *et al.*, 2024).

#### Overview of the cargo of embryo-derived EVs detected/reported

Nineteen of the 32 studies reported molecular cargo of the EVs. Thirteen of these studied the mRNA, microRNA (miRNA), DNA, and/or protein cargo without correlation with the quality of the embryo from which the EVs originated (Table 2). Two of these studies were of human EVs; one examined four pluripotency-related gene transcripts (Giacomini *et al.*, 2017), and the other assessed total RNA transcripts (Makieva *et al.*, 2024). However, no overlapping transcripts were identified. Of the bovine studies, two assessed proteins from 8- to 16-cell or blastocyst-stage embryos, with 61 proteins overlapping between the two studies (Supplementary Table S3) (Mazzarella *et al.*, 2025a,b). Meanwhile, another bovine study reported three full-length mRNA transcripts associated with EVs released from morula-stage embryos (Townsend *et al.*, 2025). None of the mRNA transcripts identified in the bovine study overlapped with the mRNA identified in the human studies. Four bovine studies demonstrated that EV cargoes varied according to the developmental stage of the embryos (Melo-Báez *et al.*, 2023), the oxygen concentration of the culture conditions (Taqi *et al.*, 2019), and the mode of embryo production, i.e. whether *in vitro* or *in vivo* produced (Bridi *et al.*, 2021; De Bem *et al.*, 2024). Three of the bovine studies assessed miRNA cargo in *in vitro*- and/or *in vivo*-produced embryo EVs, identifying 112, 393, or 140 miRNAs depending on the study (Bridi *et al.*, 2021; Melo-Báez *et al.*, 2023; De Bem *et al.*, 2024). 29 miRNAs overlapped between all three studies, whilst 126 overlapped between two of the three studies (Supplementary Table S4).

Within the murine (Simon *et al.*, 2020; Nagy *et al.*, 2025), porcine (Saadeldin *et al.*, 2014), and ovine (Burns *et al*., 2016) studies assessing embryo EV cargo, they assessed DNA, miRNA, mRNA, and proteins. In the murine study, which assessed miRNA cargo, all 11 miRNAs identified within the manuscript (mmu-mir-30e-5p, mmu-mir-362-5p, mmu-mir-223-3p, mmu-mir-340-5p, mmu-mir-30c-5p, mmu-mir-29a-3p, mmu-mir-532-5p, mmu-mir-503-5p, mmu-mir-210-3p, mmu-mir-375-3p, and mmu-mir-29a-3p) overlapped with equivalent miRNAs identified in the bovine studies (Supplementary Tables S4 and S5). Together, these studies provide supporting evidence that mammalian EVs contain several different biological products.

#### The association between embryo EV concentration and size and embryo developmental competence

##### Human

Two human studies investigated the relationship between EV concentration and size with embryo quality (Table 3). One study, which collected EVs during the blastulation period (Days 3–5), found higher EV concentrations from euploid embryos compared to aneuploid embryos, whilst no association with EV size was found (Makieva *et al.*, 2024). In contrast, the other study, which sampled EVs released during the zygote to morula stage (Days 1–3), reported no clear association between EV concentration and embryo quality, as evaluated by blastomere number and morphology. However, the authors did show a larger EV size (3–10 nm increase) was associated with morphologically high-quality embryos (Veraguas *et al.*, 2021).

Only one human study (Abu-Halima *et al.*, 2017) investigated the association between embryo EV concentration or size with clinical outcomes. This study sampled EVs from the zygote to morula or blastocyst stage of development (Days 1–3 or 1–5) and showed a lower EV concentration was associated with pregnancy; however, they had a very small sample size (n = 4 positive pregnancies and n = 4 negative pregnancies). Within the same study, the authors also investigated the relationship between EV size and pregnancy outcomes and found the release of larger EVs (148.8 nm versus 141.7 nm) was associated with pregnancy (Abu-Halima *et al.*, 2017). However, the data presented were from only one positive pregnancy and one negative pregnancy sample. To date, there are no studies reporting the relationship between EV characteristics and live birth.

In humans, there is currently inadequate evidence to draw conclusions regarding the association of embryo EV concentration or size with embryo quality or pregnancy. With the certainty of the evidence being very low according to the quality of evidence GRADE assessment (Supplementary Table S2).

##### Bovine

Eight bovine studies examined the association between EV concentration and embryo quality (Table 3). Two studies found that lower EV concentration was associated with morphologically high-quality embryos (Dissanayake *et al.*, 2020; Melo-Báez *et al.*, 2020). One of these studies (Melo-Báez *et al.*, 2020) sampled EVs during the compaction period (Days 3.5–5); while the other study (Dissanayake *et al.*, 2020) assessed three developmental windows, observing lower EV concentration associated with high-quality embryos when EVs were sampled during the zygote to four-cell (Days 1–2) and zygote to blastocyst stage (Days 1–8), but not from the zygote to morula stage (Days 1–5). Contrary to these two studies, Pavani *et al*. reported a higher EV concentration in high-quality embryos released during the zygote to blastocyst stage (Days 1–8). However, the study sample size was small—n = 3 (pools of n = 26 samples in each) (Pavani *et al.*, 2022). The remaining five studies, sampling EVs across developmental stages, found no consistent association between EV concentration and embryo quality (Mellisho *et al.*, 2017, 2019; Dissanayake *et al.*, 2021; Caamaño *et al.*, 2024; Hamdi *et al.*, 2024).

Seven bovine studies investigated the relationship between EV size and embryo quality (Table 3). Three studies showed that larger EVs were associated with good-quality embryos (Dissanayake *et al.*, 2020; Pavani *et al.*, 2022; Hamdi *et al.*, 2024). Sample sizes for these three papers ranged from 3 to 25, with EVs sampled from zygote to four-cell, morula and blastocyst stage (Days 1–2, 1–5, and 1–8), and during the early cleavage stage (Days 2–3). In contrast, one study examined EVs released during the compaction period (Days 3.5–5) and showed that smaller EVs were associated with higher-quality embryos (those that developed to the blastocyst stage) compared to those that arrested at the morula stage (Melo-Báez *et al.*, 2020). This study had the largest sample size among all included studies, with 154 high-quality and 250 low-quality embryos included. The remaining three bovine studies that investigated this outcome had sampled EVs from the blastulation (Days 5–7/7.5) and hatching blastocyst stage (Days 7–9), and found no consistent associations between EV size and embryo quality (Mellisho *et al.*, 2017, 2019; Caamaño *et al.*, 2024).

Overall, the current evidence suggests neither concentration, nor the size, of EVs from bovine embryos have any consistent association with embryo quality, with very low certainty of evidence based on our GRADE assessment (Supplementary Table S2). The association of embryo EV concentration and size with embryo quality was not assessed in the murine, ovine, or porcine studies.

#### The association between embryo EV cargo and embryo developmental competence

##### Human

Two human studies studied the relationship between EV cargo and embryo quality (Table 4). EVs were sampled during the zygote to morula stage (Days 1–3) for both studies. The first study investigated copy number variation using array-based chromosomal genomic hybridization, comparing EVs from high-quality embryos and arrested embryos (Veraguas *et al.*, 2021). There was no difference in the EV aneuploidy rates between high-quality (50–66.6%) and arrested (57.1%) embryos; however, when comparing samples from EVs and the arrested embryos from which they were derived, the EVs had higher numbers of chromosomal abnormalities compared to the matched embryos. The other human study (Makieva *et al.*, 2024) identified specific RNA fragments from the EVs that were associated with aneuploid or euploid embryos. Four of these RNA fragments were identified as potential biomarkers. *PPM1J*, *LINC00561*, and *ANKRD34C* had decreased abundance in non-viable, aneuploid embryo EVs. While *TMED10* had increased abundance in non-viable, aneuploid embryo EVs.

However, the evidence is currently very limited and uncertain for the association of embryo EV cargo and embryo quality in humans (Supplementary Table S2).

##### Bovine

**Figure 3.**
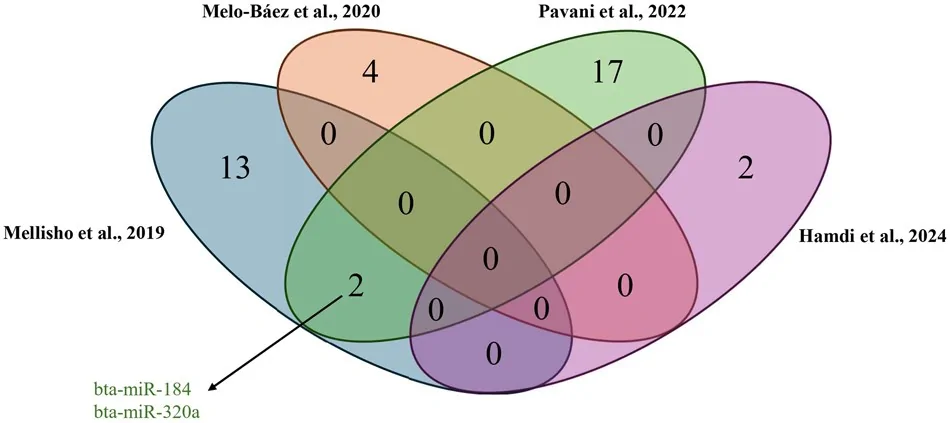
**Overlap between miRNAs identified in embryo-derived EVs to be associated with high-quality/viable embryos.** Different cut off’s were used between the four studies for assessing differently expressed miRNA: Mellisho *et al*. used log2 fold change (FC) >1.35 and <−1.35, false discovery rate (FDR) <0.05 and *P* < 0.05, whilst Melo-Báez *et al*. used log2 FC >0.5 and <−0.5 and *P* < 0.05, Pavani *et al*. used log2 FC ≥1 and ≤−1 and *P* < 0.05, and Hamdi *et al*. used FDR <10% and *P* < 0.01. Additionally, Mellisho *et al*. used counts per million (CPM) >5 for detecting miRNA exclusive to EVs from high-quality embryos. EV, extracellular vesicle; miRNA, microRNA.

For the bovine studies, five investigated EV cargo associated with embryo quality (Table 4). One study assessed the DNA concentration associated with EVs released during the blastulation period (Days 5–7) and reported decreased DNA concentration associated with high-quality embryos. However, this association was observed only in embryos produced by PA, not IVF (Caamaño *et al.*, 2024). The remaining four bovine studies (Mellisho *et al.*, 2019; Melo-Báez *et al.*, 2020; Pavani *et al.*, 2022; Hamdi *et al.*, 2024) focused on non-coding RNAs, most commonly miRNAs. However, small nucleolar RNA and transfer RNA-derived small RNA (tsRNA, also called tRNA fragments) were also studied (Mellisho *et al.*, 2019; Pavani *et al.*, 2022; Fan *et al.*, 2024b). Each study identified miRNAs that were either exclusive to or more abundant in EVs from morphologically high- or low-quality embryos (Fig. 3;  Supplementary Table S5). These studies assessed EVs from different developmental stages, including early cleavage (Days 2–3) (Hamdi *et al.*, 2024), compaction (Days 3.5–5) (Melo-Báez *et al.*, 2020), blastulation (Days 5–7.5) (Mellisho *et al.*, 2019), and the zygote to blastocyst stage (Days 1–8) (Pavani *et al.*, 2022). Only two miRNAs (bta-miR-184 and bta-miR-320a) were identified in at least two separate studies to be associated with good-quality embryo EVs (Fig. 3). Whilst only one miRNA (bta-miR-200c) that was more abundant in, or exclusive to, EVs from low-quality embryos overlapped between two studies (Supplementary Table S5).

Confusingly, miRNAs bta-miR-1 and bta-miR-10b were more abundant in high-quality embryos in one study (Mellisho *et al.*, 2019) but, in Melo Baez and Pavani studies, bta-miR-1 and bta-miR-10b, respectively, were less abundant in high-quality embryos (Melo-Báez *et al.*, 2020; Pavani *et al.*, 2022). Furthermore, Pavani *et al*. (2022) reanalysed their RNA dataset to investigate tsRNA in EVs from embryos of different qualities. This analysis found 126 tsRNAs more abundant and 22 tsRNAs less abundant in blastocyst-derived EVs compared to EVs from developmentally arrested embryos (Fan *et al.*, 2024b).

Despite this, there is currently limited evidence for an association between EV cargo and embryo quality in bovines with the certainty of evidence graded as very low (Supplementary Table S2). This outcome was not assessed in the murine, ovine, or porcine studies.

## Discussion

### Summary of key findings

This review systematically summarizes the existing literature on mammalian embryo-derived EVs and their ability to predict embryo developmental competence. The studies varied widely in EV isolation methods, sampling time points, and analytical techniques. Among the human studies, conflicting results may also be due to low sample size (<5 per group) (Abu-Halima *et al.*, 2017; Makieva *et al.*, 2024). The resulting heterogeneity makes comparisons of the studies difficult and undermines the reproducibility of the literature. The current published studies are of poor quality and lack any clinically translatable results.

In addition to the studies included in the review, a study by Pallinger *et al*. (2017) was excluded as these authors did not isolate EVs. Rather, using flow cytometry, they reported fewer propidium iodide (PI)+ EVs associated with a positive pregnancy outcome (Pallinger *et al.*, 2017). PI is a nucleic acid stain, and the results of Pallinger *et al*. (2017) suggest poor-quality embryos expel more DNA/RNA via EVs. Further investigation into EV-associated nucleic acids as a marker of embryo developmental competence is required.

Among the bovine studies, the almost absent overlap among the EV miRNA cargo, identified to be associated with embryo quality, raises an important reproducibility issue and highlights the need for studies with the same methodological design, including RNA isolation and sequencing technologies. Additionally, it is essential to note that whilst miRNA work in bovines is valuable within the bovine field, it is unknown if these results are directly translatable to humans. However, the overlap between murine miRNA and those identified in bovine miRNA studies suggests that miRNA cargo may be conserved among mammalian species. Supporting this idea, research has shown an overlap in certain miRNAs (e.g. miR-25, miR-17-5p, miR-191, miR-20a, and miR-26a) found in embryo culture media between bovines and humans (Kropp *et al.*, 2014; Capalbo *et al.*, 2016; Rio and Madan, 2021). Nevertheless, validation with human embryo-derived EV miRNA studies is required. The bovine cargo studies reported here focused primarily on miRNA, but other non-coding RNAs are also notable. For example, a recent study identified differentially expressed circular RNA (circRNA) within high- and low-quality embryos. These circRNA have binding sites for miRNA and tsRNA previously identified in embryo EVs, potentially altering their regulatory processes (Pavani *et al.*, 2022; Fan *et al.*, 2024a,b). Therefore, other cargo, such as circRNA, which are expressed by the embryo and therefore may be packaged into EVs, warrant further consideration.

In this review, we assessed only EVs released by the embryo into culture media, as this approach is both necessary and appropriate for biomarker research. However, it is important to recognize that implantation and the establishment of pregnancy is a synergistic process between the embryo and the maternal endometrium, which also secretes factors, including EVs, that may influence embryo development and promote or inhibit implantation (Segura-Benítez *et al.*, 2022, 2023; Beal *et al.*, 2023). Therefore, whilst analysing only embryo-secreted EVs is appropriate for biomarker studies, functional and clinical studies must also consider the endometrial component. This broader concept is explored in several publications assessing the co-culture of embryos and endometrial cells (Parks *et al.*, 2018; Hamdi *et al.*, 2024; Bridi *et al.*, 2025). Reporting and controlling for conditions with disrupted endometrial function is also important when investigating embryo EVs as a biomarker for clinical outcomes (Li *et al.*, 2022).

Surprisingly, most studies, regardless of the species investigated, reported the isolation of small EVs (<200 nm) (Welsh *et al.*, 2024). This was unexpected in studies where crude/unprocessed SCM was assessed (Makieva *et al.*, 2024), and studies assessing EVs that were anticipated to be apoptotic bodies (large EVs) based on the EV isolation procedures (Veraguas *et al.*, 2021). Possibly this is due to the zona pellucida only allowing the passage of small EVs to the media (Vyas *et al*., 2019). Alternatively, it may be that embryos produce extremely few large EVs. Examining EVs from intact and zona-free embryos would address this issue. In line with the MISEV guideline recommendations, future studies should refrain from using nomenclature specifying biogenesis pathways of EVs, such as exosomes or apoptotic bodies, as the apoptotic bodies identified in one study (Veraguas *et al.*, 2021) were the size of nanovesicles, while apoptotic bodies are much larger, suggesting that those EVs were not apoptotic bodies (Welsh *et al.*, 2024).

One factor that was not considered in any of the studies was contamination of the embryonic EVs by EVs from other sources, such as sperm, cumulus cells, or embryologist skin, which may also be found in SCM (Hammond *et al.*, 2017). Identifying and eliminating such contaminating EVs will be challenging and may require high-sensitivity techniques such as Raman spectroscopy for detection and very selective techniques such as affinity isolation to remove contaminants (Xu *et al.*, 2009; Lo *et al.*, 2020; Kowalska *et al.*, 2024; Zheng *et al.*, 2025).

### Strengths and limitations

This is the first systematic analysis of embryo-derived EVs as biomarkers of embryo developmental competence. It consolidates and summarizes current findings and aims to guide future high-quality research within this field.

Whilst vote counting by statistical significance is inappropriate, vote counting using direction of effect is also unreliable, and therefore, in future, standardized methodologies and robust statistical reporting must be included to enable meta-analysis (McKenzie and Brennan, 2024).

Current systematic review methodology was primarily developed to critically summarize randomized controlled trial evidence in clinical research (Higgins *et al.*, 2024b). As a result, evaluating RoB in non-clinical studies is challenging. At the time this review was initiated, no validated RoB tools existed specifically for laboratory-based studies, so we pre-specified the use of an adapted NOS scale. Although the NOS tool has some noted limitations (Van Wely *et al.*, 2025), all evidence presented in this review was ultimately downgraded due to serious or very serious concerns for RoB (Supplementary Table S2). We therefore consider this approach to provide a conservative and appropriately cautious interpretation of the potential biases affecting the included studies.

Lastly, there are relatively few studies examining EVs from human studies and whether results from animal models can be translated to humans is not clear.

### Recommendations for future research

In order to improve the quality of evidence and comparability of studies, we make the following recommendations for future research aiming to determine whether embryo-derived EVs are good biomarkers of embryo competence.

First, future studies should use appropriate methodology to isolate and characterize EVs, aligning with the MISEV guidelines when possible (Welsh *et al.*, 2024). One particularly problematic issue with characterization of the EVs was that EV counts were often performed on enriched EVs that were in volumes of buffer that were different to the volume of medium from which the EVs were isolated, without a correction for this volume change. Future studies should correct EV counts for such volume changes. Additionally, characterization should be performed only on individually cultured embryos to produce comparable results; therefore, pooling EVs or group culture of embryos is discouraged for embryo EV biomarker research.

Second, it is likely that embryos release different EVs/cargoes at different developmental stages/periods (Melo-Báez *et al.*, 2023). Therefore, it is essential that the stage of embryonic development from which EVs were sampled is reported in detail and standardized. Additionally, future work investigating the EV dynamics across developmental time periods would help progress this field.

Third, the concentration and size of particles/EVs in the medium that has not been exposed to embryos should be included as a control and reported, as this medium can contain abundant particles, which would affect the reported EV concentration and size measurements, and this will vary depending on the medium used (Giacomini *et al.*, 2017; Marin, 2019).

Fourth, future studies should use clinical outcomes as their primary endpoint for quality assessment, ideally live birth, although secondary endpoints such as blastocyst quality and aneuploidy status should be included, particularly when associating characteristics of embryo-derived EVs with miscarriage rates. Morphological quality should not be the primary endpoint because assessment of embryos is a subjective metric (Storr *et al.*, 2017) and often a poor predictor of a healthy live birth (Capalbo *et al.*, 2014). The current association with morphological quality is suboptimal, and the field will not progress until appropriate clinical developmental competence markers are thoroughly investigated.

Fifth, a major limitation of the existing studies is the small sample size. With the increasing use of IVF/ICSI worldwide, there are vast numbers of spent embryo culture medium samples generated (Ferraretti *et al.*, 2017; Kotevski *et al.*, 2025). These human samples are readily accessible and offer the possibility of studies of embryo-derived EVs with very large sample sizes. The investigation of EVs from large numbers of embryos may also allow the use of machine learning techniques to interrogate features of the EVs, not detectible by traditional methods, that can be used to predict clinical outcomes (Shamout *et al*., 2021). It will be important for future studies to include analysis of EVs from much larger numbers of embryos.

Lastly, while animal models are often necessary in biomedical research due to inaccessibility of tissues/fluids, that is not the case for human embryo-derived EVs. Given that there are differences in embryonic development and implantation between species, it is essential that, if embryo-derived EVs are to be used as biomarkers of human embryo developmental competence, studies must focus on investigating human embryo-derived EVs.

## Conclusions

This systematic review and narrative analysis expose the current lack of evidence for an association between EV concentration, size, and cargo with embryo developmental competence. This is due to the poor quality of current evidence. Therefore, analysis of EV characteristics is currently far from ready for clinical use as an embryo selection tool during IVF/ICSI. Nevertheless, while the current evidence is of poor quality, EVs are being developed as minimally invasive biomarkers of many disease states, and the poor quality of existent evidence does not mean EVs cannot be developed as biomarkers of embryo potential. In addition to our critique of the literature, we have provided recommendations for future research to improve the quality of research into EVs as biomarkers of embryo developmental competence with the aim of moving the field towards the development of clinically useful EV-based biomarkers.

## Supplementary Material

dmag014_Supplementary_Data

## Data Availability

Data used to formulate this review are available in the article and supplementary files and tables.
