# Supplementary material for: Embryo-derived extracellular vesicles and their potential as biomarkers for embryo quality during IVF: a systematic review
Source: Hum Reprod Update. 2026 May 21;32(5):613–36. doi: 10.1093/humupd/dmag014 (PMC13538716; doi:10.1093/humupd/dmag014)
Supplement: Supplementary file 1 [file dmag014_supplementary_data.pdf]

Manuscript Title: Embryo-Derived Extracellular Vesicles and their Potential as Biomarkers for Embryo Quality during IVF: A Systematic Review

Authors: Genevieve Boom[1], Vanessa Jordan[1], Lawrence W. Chamley[1], Lynsey Cree[1,2]

Affiliations: [1]Department of Obstetrics, Gynaecology, and Reproductive Sciences, The University of Auckland, Auckland, New Zealand, 1023; [2]Fertility Associates, Auckland, New Zealand, 1051

Supplementary Data File S1. Search strategies used

Supplementary Data File S2. Risk of Bias Tool adapted from Newcastle-Ottawa Scale

Supplementary Table S1. Risk of Bias Assessment of Studies

Supplementary Table S2 GRADE Assessment of Outcomes

Supplementary Table S3. Bovine EV proteins produced by in vitro embryos

Supplementary Table S4. Bovine EV miRNAs produced by in vivo or in vitro embryos

Supplementary Table S5. Bovine EV miRNAs associated with embryo quality

Supplementary Data File S1: Search strategies used

MEDLINE:

1 blastomeres/ or embryo, mammalian/ or morula/
2 (embryo* or blastoc* or trophectoderm or trophoblast* or morula* or blastomere*).ab,fx,kf,ti.
3 1 or 2
4 Extracellular Vesicles/
5 ("extracellular vesicle*" or microparticle* or exosome* or micro?vesicle* or nano?vesicle* or macro?vesicle* or nanoparticle* or "syncytial nuclear aggregate*" or "shedding vesicle*" or "membrane vesicle*" or "budding vesicle*" or "blebbing vesicle*" or "apoptotic bleb*" or "apoptotic bod*" or "extracellular bod*" or exovesicle* or migrasome* or oncosome*).ab,fx,kf,ti.
6 4 or 5
7 3 and 6

EMBASE:

1 preimplantation embryo/
2 human embryo/
3 embryo/
4 mammalian embryo/
5 blastocyst/
6 morula/
7 (embryo* or blastoc* or trophectoderm or trophoblast* or morula* or blastomere*).ab,fx,kf,ti.
8 1 or 2 or 3 or 4 or 5 or 6 or 7
9 exosome/
10 ("extracellular vesicle*" or microparticle* or exosome* or micro?vesicle* or nano?vesicle* or macro?vesicle* or nanoparticle* or "syncytial nuclear aggregate*" or "shedding vesicle*" or "membrane vesicle*" or "budding vesicle*" or "blebbing vesicle*" or "apoptotic bleb*" or "apoptotic bod*" or "extracellular bod*" or exovesicle* or migrasome* or oncosome*).ab,fx,kf,ti.
11 9 or 10
12 8 and 11

BIOSIS previews:

1 Embryo/
2 (embryo* or blastoc* or trophectoderm or trophoblast* or morula* or blastomere*).ab,cc,ti.
3 1 or 2
4 ("extracellular vesicle*" or microparticle* or exosome* or micro?vesicle* or nano?vesicle* or macro?vesicle* or nanoparticle* or "syncytial nuclear aggregate*" or "shedding vesicle*" or "membrane vesicle*" or "budding vesicle*" or "blebbing vesicle*" or "apoptotic bleb*" or "apoptotic bod*" or "extracellular bod*" or exovesicle* or migrasome* or oncosome*).ab,cc,ti.
5 3 and 4

Web of science core collection:

1 TS=(embryo* or blastoc* or trophectoderm or trophoblast* or morula* or blastomere*)
2 TI=(embryo* or blastoc* or trophectoderm or trophoblast* or morula* or blastomere*)
3 AB=(embryo* or blastoc* or trophectoderm or trophoblast* or morula* or blastomere*)

4 KP=(embryo* or blastoc* or trophectoderm or trophoblast* or morula* or blastomere*)
5 SO=(embryo* or blastoc* or trophectoderm or trophoblast* or morula* or blastomere*)
6 #1 OR #2 OR #3 OR #4 OR #5
7 TS=("extracellular vesicle*" or microparticle* or exosome* or micro$vesicle* or nano$vesicle* or macro$vesicle* or nanoparticle* or "syncytial nuclear aggregate*" or "shedding vesicle*" or "membrane vesicle*" or "budding vesicle*" or "blebbing vesicle*" or "apoptotic bleb*" or "apoptotic bod*" or "extracellular bod*" or exovesicle* or migrasome* or oncosome*)
8 TI=("extracellular vesicle*" or microparticle* or exosome* or micro$vesicle* or nano$vesicle* or macro$vesicle* or nanoparticle* or "syncytial nuclear aggregate*" or "shedding vesicle*" or "membrane vesicle*" or "budding vesicle*" or "blebbing vesicle*" or "apoptotic bleb*" or "apoptotic bod*" or "extracellular bod*" or exovesicle* or migrasome* or oncosome*)
9 AB=("extracellular vesicle*" or microparticle* or exosome* or micro$vesicle* or nano$vesicle* or macro$vesicle* or nanoparticle* or "syncytial nuclear aggregate*" or "shedding vesicle*" or "membrane vesicle*" or "budding vesicle*" or "blebbing vesicle*" or "apoptotic bleb*" or "apoptotic bod*" or "extracellular bod*" or exovesicle* or migrasome* or oncosome*)
10 KP=("extracellular vesicle*" or microparticle* or exosome* or micro$vesicle* or nano$vesicle* or macro$vesicle* or nanoparticle* or "syncytial nuclear aggregate*" or "shedding vesicle*" or "membrane vesicle*" or "budding vesicle*" or "blebbing vesicle*" or "apoptotic bleb*" or "apoptotic bod*" or "extracellular bod*" or exovesicle* or migrasome* or oncosome*)
11 SO=("extracellular vesicle*" or microparticle* or exosome* or micro$vesicle* or nano$vesicle* or macro$vesicle* or nanoparticle* or "syncytial nuclear aggregate*" or "shedding vesicle*" or "membrane vesicle*" or "budding vesicle*" or "blebbing vesicle*" or "apoptotic bleb*" or "apoptotic bod*" or "extracellular bod*" or exovesicle* or migrasome* or oncosome*)
12 #7 OR #8 OR #9 OR #10 OR #11
13 #6 AND #12

Scopus:

TITLE-ABS-KEY (embryo* OR blastoc* OR trophectoderm OR trophoblast* OR morula* OR blastomere*) AND TITLE-ABS-KEY ("extracellular vesicle*" OR microparticle* OR exosome* OR micro?vesicle* OR microvesicle* OR nano?vesicle* OR nanovesicle* OR macro?vesicle* OR macrovesicle* OR nanoparticle* OR "syncytial nuclear aggregate*" OR "shedding vesicle*" OR "membrane vesicle*" OR "budding vesicle*" OR "blebbing vesicle*" OR "apoptotic bleb*" OR "apoptotic bod*" OR "extracellular bod*" OR exovesicle* OR migrasome* OR oncosome*)

Supplementary Data File S2: Risk of Bias Tool adapted from Newcastle-Ottawa Scale (Tool further adapted from (Blanchard et al., 2024).)

Categories and items	Original tool for cross-sectional studies (Herzog *et al.*, 2013)	Adapted tool for this project (modifications in *red*)
**Selection**		
1. Representativeness of the sample	a. Truly representative of the average in the target population.* (* = 1 point) (all subjects or random sampling)<br>b. Somewhat representative of the average in the target group.* (non-random sampling)<br>c. Selected group of users/convenience sample.<br>d. No description of the derivation of the included subjects.	a. Truly representative of the average in the target population (all subjects or random sampling) **(high quality)**<br>b. Somewhat representative of the average target group (non-random sampling;) **(moderate quality)**<br>c. Selected group of users/convenience sample. **(low quality)**<br>d. No description of the derivation of the included subjects. **(low quality)**
2. Sample size	a. Justified and satisfactory (including sample size calculation).*<br>b. Not justified.<br>c. No information provided.	a. Justified in a satisfactory manner (including sample size calculation).**(high quality)**<br>b. No appropriate justification but sample collected big enough to detect differences for EV concentration and size. **(moderate quality**)<br>c. No information provided **(low quality)**
3. Non-respondents/missing data	a. Proportion of target sample recruited attains pre-specified target or basic summary of non-respondent characteristics in sampling frame recorded.*<br>b. Unsatisfactory recruitment rate, no summary data on non-respondents.<br>c. No information provided.	a. Results provided for all or >95% of embryos that were studied **(high quality)**<br>b. No summary data on non-respondents. **(low quality)**<br>c. No information provided (**low quality**)

Categories and items	Original tool for cross-sectional studies (Herzog *et al.*, 2013)	Adapted tool for this project (modifications in *red*)
4. Ascertainment of the exposure (risk factor)	a. Independent verification/clinic registers/hospital records only.** (** = 2 points)<br>b. Parental or personal recall and vaccine/hospital records.*<br>c. Parental/personal recall only.	a. Gold standard (MISEV 2018 Steps of EV characterisation are met in the following areas: (1) Source of EVs and EV prep described quantitatively;<br>(2) at least one protein from categories 1-3 measured;<br>(3) at least one technique providing images of single EVs;<br>(4) at least one single particle analysis technique that estimates biophysical features of EVs) **(high quality)**<br>b. Less than gold standard (Only 2 or 3 of the 4 steps of MISEV 2018 EV characterisation met). **(moderate quality)**<br>c. Only one MISEV 2018 EV characterisation step performed. **(low quality)**<br>Not applicable to document analyses
**Comparability**		
1. Comparability of subjects in different outcome groups on the basis of design or analysis. Confounding factors controlled.	a. Data/results adjusted for relevant predictors/risk factors/confounders for example age, sex, time since vaccination, etc.**<br>b. Data/results not adjusted for all relevant confounders/risk factors/information not provided.	a. Data/results adjusted for relevant predictors/risk factors/confounders. **(high quality)**<br>b. Data/results not adjusted for all relevant confounders/risk factors/information not provided. (**low quality**)
**Outcome**		
1. Assessment of outcome	a. Independent blind assessment using objective validated laboratory methods.**<br>b. Unblinded assessment using objective validated laboratory methods.**<br>c. Used non-standard or non-validated laboratory methods with gold standard.*	a. Independent blind assessment using objective validated laboratory methods. **(high quality)**<br>b. Unblinded assessment using objective validated laboratory methods. **(high quality)**<br>c. Unblinded and could have been influenced by knowledge of embryo quality **(low quality)**<br>d. No description. **(low quality)**

**Categories and items**	**Original tool for cross-sectional studies (Herzog *et al.*, 2013)**	**Adapted tool for this project (modifications in *red*)**
	d. No description/non-standard laboratory methods used.	
2. Statistical test	a. Statistical test used to analyse the data clearly described, appropriate and measures of association presented including CIs and probability level (*p* value).*<br><br>b. Statistical test not appropriate, not described or incomplete.	a. Statistical test used to analyse the data clearly described, appropriate and measures of association presented including a measurement of error and probability level (*p* value) **(high quality)**<br><br>b. Statistical test not appropriate, not described or incomplete. **(low quality)**
**Overall rating**		
	* = 1 point<br>** = 2 points<br>**Very Good Studies**: 9–10 points<br>**Good Studies:** 7–8 points<br>**Satisfactory Studies:** 5–6 points<br>**Unsatisfactory Studies:** 0–4 points	**High quality:** 7 out of 7 items rated high<br><br>**Moderate quality:** 4 out of 7 items rated high or moderate<br><br>**Low quality:** <4 items rated as high or moderate

Supplementary Table S1: Risk of Bias Assessment of Studies

	Selection				Comparability	Outcome		
**Reference**	**Representativeness of the sample**	**Sample size**	**Non-respondents/ missing data**	**Ascertainment of the exposure (risk factor)**	**Comparability of subjects in different outcome groups on the basis of design or analysis**	**Assessment of outcome**	**Statistical test**	**Overall**
**Abu-Halima *et al.*, 2017**	(b) Moderate	(c) Low	(c) Low	(b) Moderate	(b) Low	(b) High	(b) Low	Low quality
**Aguilera *et al.*, 2023**	(b) Moderate	(c) Low	(c) Low	(b) Moderate	(b) Low	(b) High	(b) Low	Low quality
**Aguilera *et al.*, 2024**	(b) Moderate	(b) Moderate	(c) Low	(b) Moderate	(b) Low	(b) High	(b) Low	Moderate quality
**Bridi *et al.*, 2021**	(b) Moderate	(c) Low	(c) Low	(b) Moderate	(b) Low	(b) High	(a) High	Moderate quality
**Burns *et al.*, 2016**	(b) Moderate	(c) Low	(c) Low	(b) Moderate	(b) Low	(b) High	(b) Low	Low quality
**Caamano *et al.*, 2024**	(b) Moderate	(c) Low	(c) Low	(a) High	(b) Low	(b) High	(a) High	Moderate quality
**De Bem *et al.*, 2024**	(b) Moderate	(c) Low	(c) Low	(b) Moderate	(b) Low	(b) High	(b) Low	Low quality
**Dissanayake *et al.*, 2020**	(a) High	(c) Low	(c) Low	(a) High	(b) Low	(b) High	(a) High	Moderate quality
**Dissanayake *et al.*, 2021**	(a) High	(c) Low	(c) Low	(a) High	(b) Low	(b) High	(b) Low	Low quality

**Giacomini *et al.*, 2017**	(b) Moderate	(c) Low	(c) Low	(b) Moderate	(b) Low	(b) High	(a) High	Moderate quality
**Hamdi *et al.*, 2024**	(a) High	(c) Low	(c) Low	(b) Moderate	(b) Low	(b) High	(a) High	Moderate quality
**Kim *et al.*, 2019**	(b) Moderate	(c) Low	(c) Low	(c) Low	(b) Low	(c) Low	(a) High	Low quality
**Lange-Consiglio *et al.*, 2025**	(b) Moderate	(c) Low	(c) Low	(b) Moderate	(b) Low	(b) High	(a) High	Moderate quality
**Makieva *et al.*, 2024**	(b) Moderate	(c) Low	(c) Low	(b) Moderate	(b) Low	(b) High	(b) Low	Low quality
**Marin, 2019**	(b) Moderate	(c) Low	(c) Low	(b) Moderate	(b) Low	(b) High	(b) Low	Low quality
**Mazzarella *et al.*, 2025a**	(b) Moderate	(c) Low	(c) Low	(b) Moderate	(b) Low	(b) High	(a) High	Moderate quality
**Mazzarella *et al.*, 2025b**	(b) Moderate	(c) Low	(c) Low	(b) Moderate	(b) Low	(b) High	(a) High	Moderate quality
**Mellisho *et al.*, 2017**	(b) Moderate	(c) Low	(c) Low	(b) Moderate	(b) Low	(b) High	(a) High	Moderate quality
**Mellisho *et al.*, 2019**	(b) Moderate	(b) Moderate	(c) Low	(b) Moderate	(b) Low	(b) High	(a) High	Moderate quality
**Melo-Baez *et al.*, 2020**	(b) Moderate	(b) Moderate	(c) Low	(b) Moderate	(b) Low	(b) High	(b) Low	Moderate quality
**Melo-Baez *et al.*, 2023**	(b) Moderate	(b) Moderate	(c) Low	(b) Moderate	(b) Low	(b) High	(a) High	Moderate quality

**Nagy *et al.*, 2025**	(b) Moderate	(c) Low	(c) Low	(c) Low	(b) Low	(b) High	(a) High	Low quality
**Pavani *et al.*, 2019**	(a) High	(c) Low	(c) Low	(a) High	(b) Low	(b) High	(a) High	Moderate quality
**Pavani *et al.*, 2020**	(a) High	(c) Low	(c) Low	(b) Moderate	(b) Low	(b) High	(b) Low	Low quality
**Pavani *et al.*, 2022**	(a) High	(c) Low	(c) Low	(a) High	(b) Low	(b) High	(a) High	Moderate quality
**Qu *et al.*, 2017**	(a) High	(c) Low	(c) Low	(c) Low	(b) Low	(c) Low	(a) High	Low quality
**Saadeldin *et al.*, 2014**	(a) High	(c) Low	(c) Low	(c) Low	(b) Low	(b) High	(a) High	Low quality
**Simon *et al.*, 2020**	(a) High	(c) Low	(c) Low	(b) Moderate	(b) Low	(b) High	(b) Low	Low quality
**Taqi *et al.*, 2019**	(a) High	(c) Low	(c) Low	(b) Moderate	(b) Low	(b) High	(b) Low	Low quality
**Townsend *et al.*, 2025**	(b) Moderate	(c) Low	(c) Low	(b) Moderate	(b) Low	(b) High	(b) Low	Low quality
**Veraguas *et al.*, 2020**	(b) Moderate	(c) Low	(c) Low	(b) Moderate	(b) Low	(b) High	(a) High	Moderate quality
**Vyas *et al.*, 2019**	(b) Moderate	(c) Low	(c) Low	(b) Moderate	(b) Low	(b) High	(b) Low	Low quality

Supplementary Table S2: GRADE Assessment of Outcomes

Certainty assessment							Certainty
**No of studies**	**Study design**	**Risk of bias**	**Inconsistency**	**Indirectness**	**Imprecision**	**Other considerations**	
The association between embryo EV concentration and embryo quality in humans							
2	non-randomised studies	serious[a]	serious[b]	not serious	very serious[c]	none	⨁◯◯◯ Very low[a,b,c]
The association between embryo EV concentration and embryo quality in bovine							
8	non-randomised studies	serious[a]	serious[b]	not serious	not serious	none	⨁◯◯◯ Very low[a,b]
The association between embryo EV size and embryo quality in human							
2	non-randomised studies	serious[a]	serious[b]	not serious	very serious[c]	none	⨁◯◯◯ Very low[a,b,c]
The association between embryo EV size and embryo quality in bovine							
7	non-randomised studies	serious[a]	serious[b]	not serious	not serious	none	⨁◯◯◯ Very low[a,b]
The association between embryo EV cargo and embryo quality in human							
2	non-randomised studies	serious[a]	not serious	not serious	very serious[c]	none	⨁◯◯◯ Very low[a,c]
The association between embryo EV cargo and embryo quality in bovine							
5	non-randomised studies	serious[a]	serious[b]	not serious	very serious[c]	none	⨁◯◯◯ Very low[a,b,c]
The association between embryo EV concentration and pregnancy/live birth rates in human							
1	non-randomised studies	very serious[d]	not serious	not serious	extremely serious[e]	none	⨁◯◯◯ Very low[d,e]
The association between embryo EV size and pregnancy/live birth rates in human							
1	non-randomised studies	very serious[d]	not serious	not serious	extremely serious[e]	none	⨁◯◯◯ Very low[d,e]

a. Downgraded one level for risk of bias based on EV characterisation; b. Downgraded one level for inconsistency due to high heterogeneity between studies; c. Downgraded two levels for imprecision as sample size less than 100 EV samples; d. Downgraded two levels for risk of bias based on EV characterisation and lack of appropriate statistical test;
e. Downgraded three levels for imprecision as samples size less than 10 EV samples

Supplementary Table S3: Bovine EV proteins produced by in vitro embryos

Protein (Accession, Symbol)	Mazzarella, Sánchez, Egido, *et al.*, 2025a	Mazzarella, Sánchez, Fernandez-Fuertes, *et al.*, 2025b
A0A3Q1M4X6, SMUG1	✓	✓
A0A3Q1MBQ7, DNAH5	✓	✓
A0A4W2BM61, PBRM1	✓	✓
A0A4W2G1A4, KRT76	✓	✓
A0A4W2HA98, CEP135	✓	✓
A0A4W2IR82, KRT20	✓	✓
E1BAU6, INPP5E	✓	✓
E1BIS6, SYNM	✓	✓
F1MWJ0, CDSN	✓	✓
Q0MRP5, LZ	✓	✓
A0A3Q1MDT7, –	✓	✓
A0A4W2D966, VPS13D	✓	✓
A0A4W2ECV0, AIDA	✓	✓
A0A4W2ENX3, KRT75	✓	✓
A0A4W2FD79, ZNF638	✓	✓
F1MIW8, DSG1	✓	✓
A0A4W2EBU0, ARG1	✓	✓
A0A3Q1M7Y8, PLOD1	✓	✓
A0A452DI79, TSN	✓	✓
A0A4W2H672, MELTF	✓	✓
A0A3Q1ML29, RANGAP1	✓	✓
A0A4W2BW17, EMILIN2	✓	✓
A0A4W2E220, FARSA	✓	✓
V6F7W7, UBE4A	✓	✓
A0A4W2E085, SRSF3	✓	✓
A0A4W2EAU0, RPS27A	✓	✓
A0A4W2H0D3, ALDH2	✓	✓
A0A3Q1LVU1, FTH1	✓	✓
A0A4W2HHA6, –	✓	✓
A0A4W2HV66, RPS16	✓	✓
P62803, –	✓	✓
A0A4W2GW83, ALB	✓	✓
A0A4W2CGL9, SMC3	✓	✓
A0A3Q1M1M7, JUP	✓	✓
A0A4W2BZQ6, XRCC6	✓	✓
A0A4W2GLG6, CACNA2D1	✓	✓

Protein (Accession, Symbol)	Mazzarella, Sánchez, Egido, *et al.*, 2025a	Mazzarella, Sánchez, Fernandez-Fuertes, *et al.*, 2025b
A0A4W2HPP0, ─	✓	✓
F1MXR0, BAIAP2L1	✓	✓
E1BPI3, FRK	✓	✓
A0A3Q1M2V8, DOCK1	✓	✓
P10096, GAPDH	✓	✓
A0A3Q1MHG7, ANXA4	✓	✓
P08728, KRT19	✓	✓
A0A4W2DYQ2, ACTB	✓	✓
G3X6I0, FCGBP	✓	✓
A0A4W2GJT0, TPM1	✓	✓
A0A4W2CVQ1, ANXA2	✓	✓
F6S1Q0, KRT18	✓	✓
F1MU12, KRT8	✓	✓
A7MBJ5, CAND1	✓	✓
A0A4W2GIZ9, ANXA6	✓	✓
A0A4W2BY01, VCL	✓	✓
A0A4W2CIB6, VIM	✓	✓
F1MF78, SYNE2	✓	✓
A0A4W2H231, DSP	✓	✓
A0A3Q1ML65, EEA1	✓	✓
A0A4W2CJT3, NUMA1	✓	✓
F1MQ37, MYH9	✓	✓
F1MYC9, SPTBN1	✓	✓
A0A4W2HR21, SPTAN1	✓	✓
E1BDX8, DYNC1H1	✓	✓
E1BIP8, SERPINB13		✓
A0A4W2D0K5, TTC26		✓
A0A4W2IP44, DNAI2		✓
A5D9E1, TPD52		✓
A0A4W2IQQ6, COG5		✓
A0A4W2D0E5, RPL36		✓
G3N156, PKP1		✓
A0A4W2E1C8, PPP2R5D		✓
A0A4W2FLQ1, PMM2		✓
A0A4W2GAE4, CLASP2		✓
A5D9B6, SDCBP		✓
G3MXV0, FKBP5		✓

**Protein (Accession, Symbol)**	**Mazzarella, Sánchez, Egido, *et al.*, 2025a**	**Mazzarella, Sánchez, Fernandez-Fuertes, *et al.*, 2025b**
Q3ZC07, ACTC1		✓
G3MWJ8, DDX3X		✓
A0A4W2E1E2, CLIP1		✓
A0A4W2CDR8, PSMA6		✓
A0A4W2FKP9, GNAQ		✓
A8E654, COL18A1		✓
A0A3Q1MCC2, AP3D1		✓
A0A4W2HG87, DDX31		✓
A0A4W2GZL4, IL1RAP		✓
A0A4W2GAK0, SYNE1		✓
F1MWD3, CCT5		✓
F1MUN7, BROX		✓
A0A4W2HAX4, TTLL12		✓
A0A4W2HEC9, RASA4B		✓
E1B8N6, LMNB1		✓
A0A088Q0A9, —		✓
G3N262, RPS3A		✓
A0A4W2C3S4, RPL3		✓
A0A4W2IHM5, PARP1		✓
A0A4W2HNU0, MYO1C		✓
A0A4W2BVA2, LAMB2		✓
E1B864, ITGB4		✓
F2FB38, MUC16		✓
Q0VCU1, ACO1		✓
A3KMV5, UBA1		✓
Q0VCX2, HSPA5		✓
G5E630, USP9X		✓
F1MYG5, LMNA		✓
A0A4W2HQR5, HUWE1		✓
A0A4W2HM59, CSPG4		✓
F1MNT4, LAMB1		✓
A0A4W2DQS5, MYH11		✓
A0A4W2F0I3, COPA		✓
A0A452DIZ9, MYH10		✓
A0A4W2DA54, HSPG2		✓
A0A3Q1MQ34, FLNB		✓
A0A4W2BU36, AHNAK		✓

Protein (Accession, Symbol)	Mazzarella, Sánchez, Egido, *et al.*, 2025a	Mazzarella, Sánchez, Fernandez-Fuertes, *et al.*, 2025b
A0A3Q1LXK9, PLEC		✓
A0A4W2CM56, –	✓	
A0A3Q1LWU2, SPTBN2	✓	
F5XVA9, VWF	✓	
A0A1L6KTE1, L2	✓	
A0A4W2CAZ6, ITFG1	✓	
F1MJ96, ARHGEF37	✓	
A0A4W2F627, HNRNPR	✓	
Q148H5, KRT71	✓	
A0A4W2IBK1, CTSD	✓	
F1N3S4, CUL4A	✓	
A0A4W2IJI4, TGM1	✓	
A0A4W2HRJ1, ACTR1B	✓	
A7E306, NID2	✓	
A1A4I8, XPO7	✓	
A0A4W2EXD9, PRDX1	✓	
A0A4W2GUV8, RPSA	✓	
J7K1V4, –	✓	
A0A4W2HN26, FLNA	✓	
F1N6D5, EFTUD2	✓	
A0A4W2H475, RPLP0	✓	
A0A4W2FJT1, HSPA8	✓	

✓ indicates the protein was identified in the embryo EV samples analysed.

Supplementary Table S4: Bovine EV miRNAs produced by in vivo or in vitro embryos

miRNA	Bridi *et al.*, 2021	Melo-Báez *et al.*, 2023	De Bem *et al.*, 2024
bta-miR-10b	✓	✓	✓
bta-miR-192	✓	✓	✓
bta-miR-194	✓	✓	✓
bta-miR-103	✓	✓	✓
bta-let-7a-5p	✓	✓	✓
bta-let-7b	✓	✓	✓
bta-let-7d	✓	✓	✓
bta-miR-125b	✓	✓	✓
bta-miR-134	✓	✓	✓
bta-miR-127	✓	✓	✓
bta-miR-143	✓	✓	✓
bta-miR-151-5p	✓	✓	✓
bta-miR-191	✓	✓	✓
bta-miR-20a	✓	✓	✓
bta-miR-22-5p	✓	✓	✓
bta-miR-221	✓	✓	✓
bta-miR-24-3p	✓	✓	✓
bta-miR-27a-3p	✓	✓	✓
bta-miR-30c	✓	✓	✓
bta-miR-30d	✓	✓	✓
bta-miR-320a	✓	✓	✓
bta-miR-375	✓	✓	✓
bta-miR-486	✓	✓	✓
bta-miR-92a	✓	✓	✓
bta-miR-92b	✓	✓	✓
bta-miR-93	✓	✓	✓
bta-miR-935	✓	✓	✓
bta-miR-1246	✓	✓	✓
bta-miR-1307	✓	✓	✓
bta-miR-584	✓	✓	
bta-miR-760-5p	✓	✓	
bta-miR-139	✓		✓
bta-miR-660	✓		✓
bta-miR-708	✓		✓
bta-miR-106a	✓		✓
bta-miR-124a	✓		✓
bta-miR-124b	✓		✓
bta-miR-126-3p	✓		✓
bta-miR-130a	✓		✓
bta-miR-130b	✓		✓
bta-miR-132	✓		✓
bta-miR-133a	✓		✓

**miRNA**	**Bridi *et al.*, 2021**	**Melo-Báez *et al.*, 2023**	**De Bem *et al.*, 2024**
bta-miR-145	✓		✓
bta-miR-154b	✓		✓
bta-miR-149-5p	✓		✓
bta-miR-16a	✓		✓
bta-miR-16b	✓		✓
bta-miR-193a-5p	✓		✓
bta-miR-187	✓		✓
bta-miR-195	✓		✓
bta-miR-197	✓		✓
bta-miR-19b	✓		✓
bta-miR-223	✓		✓
bta-miR-296-3p	✓		✓
bta-miR-296-5p	✓		✓
bta-miR-302d	✓		✓
bta-miR-31	✓		✓
bta-miR-331-5p	✓		✓
bta-miR-323	✓		✓
bta-miR-339b	✓		✓
bta-miR-34a	✓		✓
bta-miR-371	✓		✓
bta-miR-382	✓		✓
bta-miR-378	✓		✓
bta-miR-378b	✓		✓
bta-miR-411a	✓		✓
bta-miR-421	✓		✓
bta-miR-425-3p	✓		✓
bta-miR-429	✓		✓
bta-miR-4523	✓		✓
bta-miR-433	✓		✓
bta-miR-503-3p	✓		✓
bta-miR-505	✓		✓
bta-miR-494	✓		✓
bta-miR-592	✓		✓
bta-miR-664a	✓		✓
bta-miR-760-3p	✓		✓
bta-miR-665	✓		✓
bta-miR-677	✓		✓
bta-miR-874	✓		✓
bta-miR-877	✓		✓
bta-miR-1296	✓		✓
bta-miR-1249	✓		✓
bta-miR-1260b	✓		✓
bta-miR-1343-3p	✓		✓
bta-miR-1282	✓		✓
bta-miR-302b		✓	✓

miRNA	Bridi *et al.*, 2021	Melo-Báez *et al.*, 2023	De Bem *et al.*, 2024
bta-miR-96		✓	✓
bta-miR-22-3p		✓	✓
bta-miR-215		✓	✓
bta-miR-26a		✓	✓
bta-miR-29b		✓	✓
bta-miR-29c		✓	✓
bta-miR-29d-3p		✓	✓
bta-miR-2284ab		✓	✓
bta-miR-1291		✓	✓
bta-miR-2408		✓	✓
bta-miR-106b		✓	✓
bta-miR-411c-5p		✓	✓
bta-miR-3956		✓	✓
bta-miR-654		✓	✓
bta-miR-6123		✓	✓
bta-miR-154c		✓	✓
bta-miR-2889		✓	✓
bta-miR-217		✓	✓
bta-miR-181c		✓	✓
bta-miR-148a		✓	✓
bta-miR-423-5p		✓	✓
bta-miR-186		✓	✓
bta-miR-23a		✓	✓
bta-miR-27b		✓	✓
bta-miR-125a		✓	✓
bta-miR-1		✓	✓
bta-let-7c		✓	✓
bta-miR-100		✓	✓
bta-miR-99a-5p		✓	✓
bta-miR-29a		✓	✓
bta-miR-140		✓	✓
bta-miR-25		✓	✓
bta-miR-193a-3p		✓	✓
bta-let-7f		✓	✓
bta-miR-182		✓	✓
bta-miR-184		✓	✓
bta-miR-146b		✓	✓
bta-miR-99b		✓	✓
bta-miR-205		✓	✓
bta-miR-7		✓	✓
bta-miR-30e-5p		✓	✓
bta-miR-423-3p		✓	✓
bta-miR-101		✓	✓
bta-miR-365-3p		✓	✓
bta-miR-26b		✓	✓

miRNA	Bridi *et al.*, 2021	Melo-Báez *et al.*, 2023	De Bem *et al.*, 2024
bta-miR-122		✓	✓
bta-miR-146a		✓	✓
bta-miR-148b		✓	✓
bta-let-7g		✓	✓
bta-miR-502a		✓	✓
bta-miR-451		✓	✓
bta-miR-151-3p		✓	✓
bta-miR-30a-5p		✓	✓
bta-miR-484		✓	✓
bta-miR-21-5p		✓	✓
bta-miR-181a		✓	✓
bta-miR-142-5p		✓	✓
bta-miR-10a		✓	✓
bta-miR-455-5p		✓	✓
bta-miR-200c		✓	✓
bta-miR-185		✓	✓
bta-miR-2387		✓	✓
bta-let-7i		✓	✓
bta-miR-183		✓	✓
bta-miR-15b		✓	✓
bta-miR-504		✓	✓
bta-miR-222		✓	✓
bta-miR-2890		✓	✓
bta-miR-542-5p	✓		
bta-miR-149-3p	✓		
bta-miR-188	✓		
bta-miR-320b	✓		
bta-miR-346	✓		
bta-miR-453	✓		
bta-miR-489	✓		
bta-miR-541	✓		
bta-miR-615	✓		
bta-miR-631	✓		
bta-miR-574	✓		
bta-miR-656	✓		
bta-miR-669	✓		
bta-miR-763	✓		
bta-miR-767	✓		
bta-miR-940	✓		
bta-miR-1224	✓		
bta-miR-1225-3p	✓		
bta-miR-1247-3p	✓		
bta-miR-1247-5p	✓		
bta-miR-1343-5p	✓		
bta-miR-1281	✓		

miRNA	Bridi *et al.*, 2021	Melo-Báez *et al.*, 2023	De Bem *et al.*, 2024
bta-miR-1388-3p	✓		
RNT43 snoRNA	✓		
bta-miR-1287	✓		
bta-miR-380-5p	✓		
bta-miR-7863		✓	
bta-miR-2367-5p		✓	
bta-miR-2422		✓	
bta-miR-2436-5p		✓	
bta-miR-2464-3p		✓	
bta-miR-2894		✓	
bta-miR-2442		✓	
bta-miR-2900		✓	
bta-miR-6524		✓	
bta-miR-2373-5p		✓	
bta-miR-2361		✓	
bta-miR-2438		✓	
bta-miR-329b		✓	
bta-miR-2417		✓	
bta-miR-2436-3p		✓	
bta-miR-2320-5p		✓	
bta-miR-2382-3p		✓	
bta-miR-764		✓	
bta-miR-2415-3p		✓	
bta-miR-2439-5p		✓	
bta-miR-2449		✓	
bta-miR-2415-5p		✓	
bta-miR-2316		✓	
bta-miR-2368-3p		✓	
bta-miR-2307		✓	
bta-miR-2344		✓	
bta-miR-2481		✓	
bta-miR-23b-3p		✓	
bta-miR-6534		✓	
bta-miR-2478		✓	
bta-miR-363		✓	
bta-miR-9-5p		✓	
bta-miR-144		✓	
bta-miR-2418		✓	
bta-miR-2428		✓	
bta-miR-2460		✓	
bta-miR-2285ab		✓	
bta-miR-2349		✓	
bta-miR-2903		✓	
bta-miR-6121-5p		✓	
bta-miR-1248			✓

miRNA	Bridi *et al.*, 2021	Melo-Báez *et al.*, 2023	De Bem *et al.*, 2024
bta-miR-2892			✓
bta-miR-3432a			✓
bta-miR-2904			✓
bta-miR-1949			✓
bta-miR-12034			✓
bta-miR-2419-5p			✓
bta-miR-302a			✓
bta-miR-11975			✓
bta-miR-2404			✓
bta-miR-12004			✓
bta-miR-374a			✓
bta-miR-374b			✓
bta-miR-2411-5p			✓
bta-miR-17-5p			✓
bta-miR-2484			✓
bta-miR-450a			✓
bta-miR-2887			✓
bta-miR-6119-5p			✓
bta-miR-9-3p			✓
bta-miR-202			✓
bta-miR-302c			✓
bta-miR-11985			✓
bta-miR-2332			✓
bta-miR-326			✓
bta-miR-19a			✓
bta-miR-6516			✓
bta-miR-2397-5p			✓
bta-miR-199b			✓
bta-miR-2440			✓
bta-miR-3141			✓
bta-miR-107			✓
bta-miR-369-3p			✓
bta-miR-18a			✓
bta-miR-454			✓
bta-miR-652			✓
bta-miR-362-5p			✓
bta-miR-11976			✓
bta-miR-1434-5p			✓
bta-miR-2463			✓
bta-miR-210			✓
bta-miR-155			✓
bta-miR-6529b			✓
bta-miR-6529a			✓
bta-miR-3533			✓
bta-miR-11986b			✓

miRNA	Bridi *et al.*, 2021	Melo-Báez *et al.*, 2023	De Bem *et al.*, 2024
bta-miR-425-5p			✓
bta-miR-181d			✓
bta-miR-27a-5p			✓
bta-miR-1434-3p			✓
bta-miR-30b-5p			✓
bta-miR-409b			✓
bta-miR-378d			✓
bta-miR-10225a			✓
bta-miR-193b			✓
bta-miR-150			✓
bta-miR-378c			✓
bta-miR-424-3p			✓
bta-let-7e			✓
bta-miR-3596			✓
bta-miR-2285k			✓
bta-miR-6120-3p			✓
bta-miR-658			✓
bta-miR-3613a			✓
bta-miR-3613b			✓
bta-miR-206			✓
bta-miR-11991			✓
bta-miR-2424			✓
bta-miR-2318			✓
bta-miR-2320-3p			✓
bta-miR-432			✓
bta-miR-409a			✓
bta-miR-10174-3p			✓
bta-miR-365-5p			✓
bta-miR-3604			✓
bta-miR-199a-3p			✓
bta-miR-138			✓
bta-miR-2285p			✓
bta-miR-1388-5p			✓
bta-miR-128			✓
bta-miR-885			✓
bta-miR-2411-3p			✓
bta-miR-199a-5p			✓
bta-miR-7857-3p			✓
bta-miR-1271			✓
bta-miR-543			✓
bta-miR-331-3p			✓
bta-miR-503-5p			✓
bta-miR-1468			✓
bta-miR-7859			✓
bta-miR-339a			✓

miRNA	Bridi *et al.*, 2021	Melo-Báez *et al.*, 2023	De Bem *et al.*, 2024
bta-miR-4449			✓
bta-miR-6517			✓
bta-miR-191b			✓
bta-miR-196b			✓
bta-miR-2285ak-5p			✓
bta-miR-449a			✓
bta-miR-424-5p			✓
bta-miR-664b			✓
bta-miR-2336			✓
bta-miR-410			✓
bta-miR-24			✓
bta-miR-2285g			✓
bta-miR-11980			✓
bta-miR-30f			✓
bta-miR-28			✓
bta-miR-126-5p			✓
bta-miR-34b			✓
bta-miR-34c			✓
bta-miR-330			✓
bta-miR-212			✓
bta-miR-199c			✓
bta-miR-340			✓
bta-miR-2898			✓
bta-miR-3431			✓
bta-miR-361			✓
bta-miR-500			✓
bta-miR-141			✓
bta-miR-2899			✓
bta-miR-200b			✓
bta-miR-2285o			✓
bta-miR-1843			✓
bta-miR-142-3p			✓
bta-miR-2443			✓
bta-miR-2288			✓
bta-miR-129-5p			✓
bta-miR-29d-5p			✓
bta-miR-196a			✓
bta-miR-345-3p			✓
bta-miR-379			✓
bta-miR-135a			✓
bta-miR-190b			✓
bta-miR-129			✓
bta-let-7a-3p			✓
bta-miR-2450a			✓
bta-miR-98			✓

miRNA	Bridi *et al.*, 2021	Melo-Báez *et al.*, 2023	De Bem *et al.*, 2024
bta-miR-381			✓
bta-miR-9851			✓
bta-miR-2398			✓
bta-miR-2285aa			✓
bta-miR-2284x			✓
bta-miR-495			✓
bta-miR-194b			✓
bta-miR-335			✓
bta-miR-6531			✓
bta-miR-10174-5p			✓
bta-miR-32			✓
bta-miR-10225b			✓
bta-miR-4657			✓
bta-miR-194b-3p			✓
bta-miR-2285av			✓
bta-miR-2427			✓
bta-miR-147			✓
bta-miR-328			✓
bta-miR-769			✓
bta-miR-218			✓
bta-miR-369-5p			✓
bta-miR-26c			✓
bta-miR-6523a			✓
bta-miR-6523b			✓
bta-miR-204			✓
bta-miR-2483-5p			✓
bta-miR-1839			✓
bta-miR-2285b			✓
bta-miR-1298			✓
bta-miR-21-3p			✓
bta-miR-744			✓
bta-miR-431			✓
bta-miR-374c			✓
bta-miR-7857-5p			✓
bta-miR-148d			✓
bta-miR-4286			✓
bta-miR-12023			✓
bta-miR-2285u			✓
bta-miR-370			✓
bta-miR-3578			✓
bta-miR-450b			✓
bta-miR-2284y			✓
bta-miR-2311			✓
bta-miR-758			✓
bta-miR-1777a			✓

miRNA	Bridi *et al.*, 2021	Melo-Báez *et al.*, 2023	De Bem *et al.*, 2024
bta-miR-532			✓
bta-miR-2284z			✓
bta-miR-200a			✓
bta-miR-493			✓
bta-miR-129-3p			✓
bta-miR-3120			✓
bta-miR-487b			✓
bta-miR-2448-3p			✓
bta-miR-499			✓
bta-miR-95			✓
bta-miR-2376			✓
bta-miR-452			✓
bta-miR-9			✓
bta-miR-455-3p			✓
bta-miR-2285z			✓
bta-miR-2285q			✓
bta-miR-342			✓
bta-miR-136			✓
bta-miR-11972			✓
bta-miR-181b			✓
bta-miR-219-3p			✓
bta-miR-214			✓
bta-miR-1306			✓
bta-miR-3600			✓
bta-miR-152			✓
bta-miR-148c			✓
bta-miR-10164-3p			✓
bta-miR-485			✓
bta-miR-11983			✓
bta-miR-2285bc			✓
bta-miR-6526			✓
bta-miR-507b			✓
bta-miR-224			✓
bta-miR-1842			✓
bta-miR-2285au			✓
bta-miR-30b-3p			✓
bta-miR-2285ba			✓
bta-miR-7180			✓
bta-miR-2346			✓
bta-miR-380-3p			✓
bta-miR-2382-5p			✓
bta-miR-2284ac			✓
bta-miR-383			✓
bta-miR-2399-5p			✓
bta-miR-2285t			✓

**miRNA**	**Bridi *et al.*, 2021**	**Melo-Báez *et al.*, 2023**	**De Bem *et al.*, 2024**
bta-miR-671			✓
bta-miR-2431-3p			✓
bta-miR-2284h-5p			✓
bta-miR-2447			✓
bta-miR-507-3p			✓
bta-miR-7860			✓
bta-miR-15a			✓
bta-miR-2315			✓
bta-miR-324			✓
bta-miR-2885			✓
bta-miR-2450b			✓
bta-miR-11987			✓
bta-miR-6119-3p			✓
bta-miR-2323			✓

✓ indicates the miRNA was identified in the embryo EV samples analysed.

Supplementary Table S5: Bovine EV miRNAs associated with embryo quality

miRNA	Mellisho *et al.*, 2019	Melo-Baez *et al.*, 2020	Pavani *et al.*, 2022	Hamdi *et al.*, 2024
bta-miR-10a	↑			
bta-miR-10b	↑		↓	
bta-miR-1246	↑			
bta-miR-320a	↑		↑	
bta-miR-1	↑↑	↓		
bta-miR-2889	↑↑			
p-bta-miR-71	↑↑			
bta-miR-7863	↑			
bta-miR-184	↑↑		↑	
bta-miR-2368-3p	↑↑			
bta-miR-2449	↑			
bta-miR-2284ab	↑			
bta-miR-92a		↑		
bta-miR-140		↑		
bta-miR-2285av		↑		
bta-miR-222		↑		
bta-miR-11986b			↑	
bta-miR-1468			↑	
bta-miR-151-3p			↑	
bta-miR-151-5p			↑	
bta-miR-186			↑	
bta-miR-192			↑	
bta-miR-2284z			↑	
bta-miR-25			↑	
bta-miR-28			↑	
bta-miR-30e-5p			↑	
bta-miR-3432a			↑	
bta-miR-371			↑	
bta-miR-378			↑	
bta-miR-411a			↑	
bta-miR-423-5p			↑	
bta-miR-665			↑	
bta-miR-92b			↑	
miR-143				↑
miR-7975				↑
bta-miR-2316	↓			
bta-miR-3956	↓			
p-bta-miR-14	↓			
bta-miR-200c	↓			↓↓
bta-miR-2382-3p	↓			
p-bta-miR-26	↓			
p-bta-miR-61	↓			
bta-miR-2334	↓			

miRNA	Mellisho *et al.*, 2019	Melo-Baez *et al.*, 2020	Pavani *et al.*, 2022	Hamdi *et al.*, 2024
bta-miR-331-3p	↓			
bta-miR-6535	↓			
bta-miR-6521	↓			
bta-miR-425-5p	↓			
bta-miR-2483-5p	↓			
bta-miR-2325c	↓			
bta-miR-124a_bta-miR-124b	↓			
bta-miR-103		↓		
bta-miR-100		↓		
bta-miR-502a		↓		
bta-miR-146b			↓	
bta-miR-125b	↑↑			
bta-miR-2417	↑↑			
p-bta-miR-124	↑↑			

↑↑ = exclusive expression in viable embryos; ↑ = upregulated expression in viable embryos / downregulated expression in non-viable embryos; ↓↓ = exclusive expression in non-viable embryos; ↓ = upregulated expression in non-viable embryos / downregulated expression in viable embryos

References:

Abu-Halima M, Häusler S, Backes C, Fehlmann T, Staib C, Nestel S, Nazarenko I, Meese E, Keller A. Micro-ribonucleic acids and extracellular vesicles repertoire in the spent culture media is altered in women undergoing In Vitro Fertilization. *Sci Rep* 2017:**7**;1–11.

Aguilera C, Velásquez AE, Gutierrez-Reinoso MA., Wong YS, Melo-Baez B, Cabezas J, Caamaño D, Navarrete F, Rojas D, Riadi G et al. Extracellular Vesicles Secreted by Pre-Hatching Bovine Embryos Produced In Vitro and In Vivo Alter the Expression of IFNtau-Stimulated Genes in Bovine Endometrial Cells. *Int J Mol Sci* 2023:**24**;7438.

Aguilera C, Wong YS, Gutierrez-Reinoso MA, Velásquez AE, Melo-Báez B, Cabezas J, Caamaño D, Navarrete F, Castro FO, Rodriguez-Alvarez LL. Embryo-maternal communication mediated by extracellular vesicles in the early stages of embryonic development is modified by in vitro conditions. *Theriogenology* 2024:**214**;43–56.

Blanchard L, Ray S, Law C, Vega-Sala MJ, Bidonde J, Bridge G, Egan M, Petticrew M, Rutter H, Knai C. The effectiveness, cost-effectiveness and policy processes of regulatory, voluntary and partnership policies to improve food environments: an evidence synthesis. *Public Health Res* 2024:**8**;1-173.

Bridi A, Andrade GM, Del Collado M, Sangalli JR, de Ávila ACFCM, Motta IG, da Silva JCB, Pugliesi G, Silva LA, Meirelles FV et al. Small extracellular vesicles derived from in vivo- or in vitro-produced bovine blastocysts have different miRNAs profiles—Implications for embryo-maternal recognition. *Mol Reprod Dev* 2021:**88**;628–643.

Burns GW, Brooks KE, Spencer TE. Extracellular vesicles originate from the conceptus and uterus during early pregnancy in sheep. *Biol Reprod* 2016:**94**;1–11.

Caamaño D, Cabezas J, Aguilera C, Martinez I, Wong YS, Sagredo DS, Ibañez B, Rodriguez S, Castro FO, Rodriguez-Alvarez L. DNA Content in Embryonic Extracellular Vesicles Is Independent of the Apoptotic Rate in Bovine Embryos Produced In Vitro. *Animals* 2024:**14**;1041.

De Bem THC, Bridi A, Tinning H, Sampaio RV, Malo-Estepa I, Wang D, Vasconcelos EJR, Nociti RP, de Ávila ACFCM, Rodrigues Sangalli J. Biosensor capability of the endometrium is mediated in part, by altered miRNA cargo from conceptus-derived extracellular vesicles. *FASEB J* 2024:**38**;1–18.

Dissanayake K, Nõmm M, Lättekivi F, Ord J, Ressaissi Y, Godakumara K, Reshi QUA, Viil J, Jääger K, Velthut-Meikas A et al. Oviduct as a sensor of embryo quality: deciphering the extracellular vesicle (EV)-mediated embryo-maternal dialogue. *J Mol Med* 2021:**99**;685–697.

Dissanayake K, Nõmm M, Lättekivi F, Ressaissi Y, Godakumara K, Lavrits A, Midekessa G, Viil J, Bæk R, Jørgensen MM et al. Individually cultured bovine embryos produce extracellular vesicles that have the potential to be used as non-invasive embryo quality markers. *Theriogenology* 2020:**149**;104–116.

Giacomini E, Vago R, Sanchez AM, Podini P, Zarovni N, Murdica V, Rizzo R, Bortolotti D, Candiani M, Viganò P. Secretome of in vitro cultured human embryos contains extracellular vesicles that are uptaken by the maternal side. *Sci Rep* 2017:**7**;1–13.

Hamdi M, Sánchez JM, Fernandez-Fuertes B, Câmara DR, Bollwein H, Rizos D, Bauersachs S, Almiñana C. Oviductal extracellular vesicles miRNA cargo varies in response to embryos and their quality. *BMC Genomics* 2024:**25**;1–25.

Herzog R, Álvarez-Pasquin MJ, Díaz C, Del Barrio JL, Estrada JM, Gil A. Are healthcare workers' intentions to vaccinate related to their knowledge, beliefs and attitudes? a systematic review. *BMC Public Health* 2013:**1**;154.

Kim J, Lee J, Lee TB, Jun JH. Embryotrophic effects of extracellular vesicles derived from outgrowth embryos in pre- and peri-implantation embryonic development in mice. *Mol Reprod Dev* 2019:**86**;187–196.

Lange-Consiglio A, Giulia G, Paola G, Giampaolo B, Pietro R, Fausto C. LPS-Stressed Bovine Endometrial Cells upon Morulae in a Transwell Model of Embryo—Maternal Talk. *Animals* 2026:**16**;1–19.

Makieva S, Giacomini E, Scotti GM, Lazarevic D, Pavone V, Ottolina J, Bartiromo L, Schimberni M, Morelli M, Alteri A. Extracellular vesicles secreted by human aneuploid embryos present a distinct transcriptomic profile and upregulate MUC1 transcription in decidualised endometrial stromal cells. *Hum Reprod Open* 2024:**2024**;hoae014.

Marin D. Biomarkers in Reproductive Medicine: Toward Selecting the Best IVF Embryo for Transfer. *ProQuest ETD Collection - Thomas Jefferson University* 2019;AAI27542408.

Mazzarella R, Sánchez JM, Egido SG, McDonald M, Álvarez-Barrientos A, González E, Falcón-Pérez JM, Azkargorta M, Elortza F, González ME, Lonergan P, Rizos D, Fernandez-Fuertes B. Deciphering the Dialogue between the Bovine Blastocyst and the Uterus: Embryo-Induced Alterations in Extracellular Vesicle Protein Content from an Ex Vivo Model and the in Vivo Environment. *J Anim Sci Biotechnol* 2025a:**16**;1–28.

Mazzarella R, Sánchez JM, Fernandez-Fuertes B, Egido SG, McDonald M, Álvarez-Barrientos A, González E, Falcón-Pérez JM, Azkargorta M, Elortza F, González ME, Lonergan P, Rizos D. Embryo-Induced Changes in the Protein Profile of Bovine Oviductal Extracellular Vesicles. *Mol Cell Proteomics* 2025b:**24**;100935.

Mellisho EA, Briones MA, Velásquez AE, Cabezas J, Castro FO. Extracellular vesicles secreted during bastulation show viability of bovine embryos. *Reproduction* 2019:**158**;477–492.

Mellisho EA, Velásquez AE, Nuñez MJ, Cabezas JG, Cueto JA, Fader C, Castro FO, Rodríguez-Álvarez L. Identification and characteristics of Extracellular vesicles from bovine blastocysts produced in vitro. *PLoS ONE* 2017:**12**;1–15.

Melo-Báez B, Mellisho EA, Wong YS, Cabezas J, Caamaño D, Aguilera C, Riadi G, Castro FO, Rodriguez-Alvarez L. Bovine embryos release extracellular vesicles with differential miRNA signature during the compaction and blastulation stages. *Reprod Biol* 2023:**23**;100725.

Melo-Báez B, Wong YS, Aguilera CJ, Cabezas J, Mançanares ACF, Riadi G, Castro FO, Rodriguez-Alvarez L. Micrornas from extracellular vesicles secreted by bovine embryos as early biomarkers of developmental competence. *Int J Mol Sci* 2020:**21**;1–17.

Nagy B, Bognár Z, Csabai TJ, Fekete N, Buzás EI, Kovács AF, Szekeres-Barthó J, Pállinger E. Effects of Light Exposure during IVF: Transcriptomic Analysis of Murine Embryos and Embryo-Derived EVs. *Front Immunol* 2025:**16**;1–10.

Pavani KC, Hendrix A, Van Den Broeck W, Couck L, Szymanska K, Lin X, De Koster J, Van Soom A, Leemans B. Isolation and characterization of functionally active extracellular vesicles from culture medium conditioned by bovine embryos in vitro. *Int J Mol Sci* 2019:**20**;1–22.

Pavani KC, Lin X, Hamacher J, Van Den Broeck W, Couck L, Peelman L, Hendrix A, Van Soom A. The separation and characterization of extracellular vesicles from medium conditioned by bovine embryos. *Int J Mol Sci* 2020:**21**;2942.

Pavani KC, Meese T, Pascottini OB, Guan XF, Lin X, Peelman L, Hamacher J, van Nieuwerburgh F, Deforce D, Boel A, et al. Hatching is modulated by microRNA-378a-3p derived from extracellular vesicles secreted by blastocysts. *Proc Natl Acad Sci U.S.A.* 2022:**119**;1–12.

Qu P, Qing S, Liu R, Qin H, Wang W, Qiao F, Ge H, Liu J, Zhang Y, Cui W et al. Effects of embryo-derived exosomes on the development of bovine cloned embryos. *PLoS ONE* 2017:**12**;1–17.

Saadeldin IM, Kim SJ, Choi YB, Lee BC. Improvement of cloned embryos development by co-culturing with parthenotes: A possible role of exosomes/microvesicles for embryos paracrine communication. *Cell Reprogram* 2014:**16**;223–234.

Simon B, Bolumar D, Amadoz A, Jimenez-Almazán J, Valbuena D, Vilella F, Moreno I. Identification and characterization of extracellular vesicles and its DNA cargo secreted during murine embryo development. *Genes* 2020:**11**;203.

Taqi MO, Saeed-Zidane M, Gebremedhn S, Salilew-Wondim D, Khdrawy O, Rings F, Neuhoff C, Hoelker M, Schellander K, Tesfaye D. Sexual dimorphic expression and release of transcription factors in bovine embryos exposed to oxidative stress. *Mol Reprod Dev* 2019:**86**;2005–2019.

Townsend J, Gross N, Peng Z, Peñagaricano F, Yang Z, Ahsan N, Khatib H. The Embryonic DPPA3 Gene Stimulates the Expression of Pregnancy-Related Genes in Bovine Endometrial Cells. *J Dairy Sci.* 2025:**108**;6471–87.

Veraguas D, Aguilera C, Henriquez C, Velasquez AE, Melo-Baez B, Silva-Ibañez P, Castro F. O, Rodriguez-Alvarez L. Evaluation of extracellular vesicles and gDNA from culture medium as a possible indicator of developmental competence in human embryos. *Zygote* 2020:**29**;138–149.

Vyas P, Balakier H, Librach CL. Ultrastructural identification of CD9 positive extracellular vesicles released from human embryos and transported through the zona pellucida. *Syst Biol Reprod Med* 2019:**65**;273–280.
